# Evaluating All Potential Oral Complications of Diabetes Mellitus

**DOI:** 10.3389/fendo.2019.00056

**Published:** 2019-02-18

**Authors:** Martijn J. L. Verhulst, Bruno G. Loos, Victor E. A. Gerdes, Wijnand J. Teeuw

**Affiliations:** ^1^Department of Periodontology, Academic Centre for Dentistry Amsterdam, University of Amsterdam and Vrije Universiteit, Amsterdam, Netherlands; ^2^Department of Vascular Medicine, Amsterdam UMC, Amsterdam, Netherlands; ^3^Department of Internal Medicine, Spaarne Gasthuis, Hoofddorp, Netherlands

**Keywords:** diabetes mellitus, oral complications, hyperglycemia, insulin resistance, dyslipidemia, hypertension, immune dysfunction

## Abstract

Diabetes mellitus (DM) is associated with several microvascular and macrovascular complications, such as retinopathy, nephropathy, neuropathy, and cardiovascular diseases. The pathogenesis of these complications is complex, and involves metabolic and hemodynamic disturbances, including hyperglycemia, insulin resistance, dyslipidemia, hypertension, and immune dysfunction. These disturbances initiate several damaging processes, such as increased reactive oxygen species (ROS) production, inflammation, and ischemia. These processes mainly exert their damaging effect on endothelial and nerve cells, hence the susceptibility of densely vascularized and innervated sites, such as the eyes, kidneys, and nerves. Since the oral cavity is also highly vascularized and innervated, oral complications can be expected as well. The relationship between DM and oral diseases has received considerable attention in the past few decades. However, most studies only focus on periodontitis, and still approach DM from the limited perspective of elevated blood glucose levels only. In this review, we will assess other potential oral complications as well, including: dental caries, dry mouth, oral mucosal lesions, oral cancer, taste disturbances, temporomandibular disorders, burning mouth syndrome, apical periodontitis, and peri-implant diseases. Each oral complication will be briefly introduced, followed by an assessment of the literature studying epidemiological associations with DM. We will also elaborate on pathogenic mechanisms that might explain associations between DM and oral complications. To do so, we aim to expand our perspective of DM by not only considering elevated blood glucose levels, but also including literature about the other important pathogenic mechanisms, such as insulin resistance, dyslipidemia, hypertension, and immune dysfunction.

## Introduction

Diabetes mellitus (DM) is defined as a group of metabolic diseases characterized by hyperglycemia resulting from defects in insulin secretion, insulin action, or both (1). In 2014, the global prevalence of DM was estimated to be 9% (2), and almost 1.6 million deaths worldwide were caused directly by DM in 2015 (3). DM is also associated with high morbidity due to a broad range of complications, such as retinopathy, nephropathy, neuropathy, and cardiovascular disease (4, 5). Prevention and management of these complications have become major aspects of modern diabetes care. Besides these well-known complications, *oral* complications of DM can be expected as well (6–8). As a result, the International Diabetes Federation (IDF) published the “guideline on oral health for people with diabetes” in 2009, which encourages implementation of oral care in diabetes care (9). Knowing which oral complications can be expected, how often these occur in patients with DM, and understanding of the underlying pathogenesis is essential for a successful implementation of the guideline. The large majority of studies into oral complications still approach patients with DM from the limited perspective of elevated blood glucose levels. However, we know that there are many other pathogenic mechanisms that contribute to the development of other diabetic complications, including hyperglycemia, insulin resistance, dyslipidemia, hypertension, and immune dysfunction. In this report, we will review the literature about oral complications of DM from this broader perspective. To understand the biological mechanisms that might be involved, the pathogenic mechanisms of the classic diabetic complications are discussed first.

## Pathogenic Mechanisms of Diabetic Complications

Complications of DM can be divided into acute and chronic complications (1). Associations between acute effects of DM and oral complications have not yet been reported in the literature. Since oral complications are most likely the result of long-term effects of diabetes, the focus of this review will be on chronic complications. These complications are typically characterized by damage to the vasculature, usually grouped into microvascular and macrovascular diseases (5). Microvascular diseases include retinopathy, nephropathy and neuropathy. Macrovascular complications concern cardiovascular disease (CVD), such as coronary artery disease, cerebrovascular disease, and peripheral artery disease (10).

**Figure 1:**
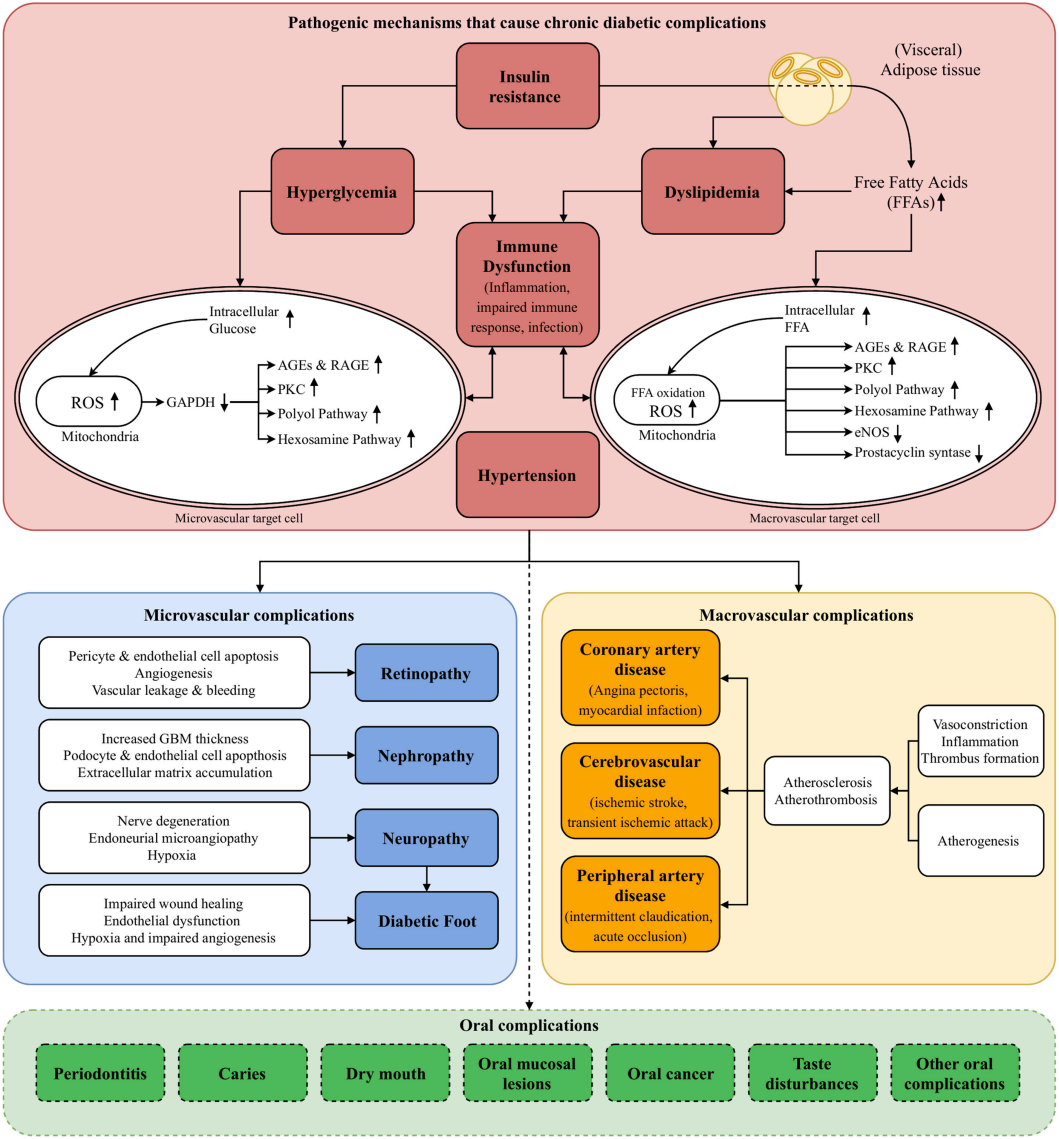
Pathogenesis of diabetic complications. The figure presents the pathogenic mechanisms of diabetes mellitus (DM; red block, section Pathogenic Mechanisms of Diabetic Complications of main text) that cause microvascular complications (blue block) and macrovascular complications (yellow block). Destruction of pancreatic B cells in T1DM and insulin resistance in T2DM result in hyperglycemia. The resulting increase of intracellular glucose in microvascular target cells, such as capillary endothelial cells, causes ROS production in the mitochondria, activating four pathogenic downstream pathways: polyol pathway, AGEs & RAGE pathway, PKC pathway, hexosamine pathway (section Hyperglycemia of main text). Especially in T2DM, insulin resistance and the abundance of (visceral) adipose tissue result in an excess flux of free fatty acids (FFAs), which are oxidized in the mitochondria of macrovascular endothelial cells. This causes activation of the same pathogenic pathways, and downregulation of protective enzymes such as eNOS and prostacyclin synthase. Pathway-selective insulin resistance also contributes to microvascular complications (section Insulin Resistance of main text). Moreover, insulin resistance and circulating FFAs result in dyslipidemia, contributing to both micro- and macrovascular complications (section Dyslipidemia of main text). Hypertension contributes to the harmful processes by activating endothelial cells, inducing a cellular inflammatory response and reducing the availability of nitric oxide, causing vasoconstriction (section Hypertension of main text). All these processes mainly exert their harmful effects by upregulation of a pro-inflammatory state at vulnerable sites. Together with an impaired immune response and consequently higher susceptibility for infections, this immune dysfunction plays a pivotal role in the development of diabetic complications (section Immune Dysfunction of main text). Possible oral complications that are discussed in this review are listed in the green block at the bottom of the figure (section Potential Oral Complications of Diabetes Mellitus of main text). eNOS, endothelial Nitric Oxide Synthase; FFAs, Free Fatty Acids; GAPDH, Glyceraldehyde 3-phosphate Dehydrogenase; GBM, Glomerular Basement Membrane; PKC, Protein Kinase C; (R)AGEs, (Receptor for) Advanced Glycation End products; ROS, Reactive Oxygen Species.

Hyperglycemia is the clinical characteristic that is used to define a patient with DM. However, several other—often intertwined—pathogenic mechanisms that characterize DM are also recognized: *insulin resistance, dyslipidemia, hypertension*, and *immune dysfunction*. In this section, we will describe how these pathogenic mechanisms are involved in the development and progression of chronic diabetic complications (Figure 1) (11). In the following sections, we will discuss possible associations between DM and oral complications by using the very same perspective each time.

### Hyperglycemia

Hyperglycemia is a key determinant for the development of complications in patients with T1DM or T2DM (12). This was demonstrated through several large trials, where intensive blood glucose control in patients with T2DM significantly reduced the risk for microvascular complications (13–18). In persons with T1DM, intensive blood glucose control also reduced the long-term risk for macrovascular complications (19), even years after the study was finished (20).

Despite the fact that each cell in the body is exposed to hyperglycemia, the damaging consequences mainly concern the endothelial cells and peripheral nerve cells (21). These target cells are not capable of maintaining a constant intracellular glucose level when blood glucose levels rise (22). The increased intracellular glucose levels trigger reactive oxygen species (ROS) production in the mitochondria. This is the *upstream* mechanism that causes inhibition of the enzyme glyceraldehyde 3-phosphate dehydrogenase (GAPDH). Consequently, four *downstream* mechanisms that are involved in tissue damage are activated: (1) increased polyol pathway flux; (2) increased non-enzymatic formation of advanced glycation end-products (AGEs) and increased expression of receptors for AGEs (RAGEs); (3) activation of protein kinase C (PKC); and (4) increased hexosamine pathway activity (21).

1. Normally, the *polyol pathway* ensures that toxic components (aldehydes) are converted into harmless inactive alcohol by an enzyme called *aldose reductase*. However, in case of hyperglycemia, aldose reductase also converts the excess intracellular glucose into sorbitol. To do so, it consumes NADPH, which is crucial for the maintenance of the intracellular antioxidant reduced glutathione (GSH). Decreased amounts of GSH cause or worsen oxidative stress, leading to cell damage or death (21, 23).
2. The non-enzymatic formation of *advanced glycation endproducts (AGEs)* results from a complex interaction between glucose and lipids, proteins or nucleic acids (24). If hyperglycemia is persistent, AGEs can accumulate in both tissue and serum, causing tissue damage through several mechanisms. They can alter intracellular proteins and thereby change cellular function (25). Also, AGEs can diffuse out of the cell and cause disruption of the signaling between the cell and its membrane, causing cell dysfunction (25). Finally, after diffusing out of the cell, they can modify circulating plasma proteins, which in turn bind to AGE receptors (e.g., RAGE) on different types of cells, such as macrophages and endothelial cells. This then induces a pro-inflammatory state, reflected by elevated levels of inflammatory cytokines in plasma, such as interleukin 6 and 1 alpha (IL-6, IL-1α) and tumor necrosis factor alpha (TNF-α) (21, 26). These processes further elicit ROS production and cause the vascular damage typical for diabetic complications (21, 23, 24, 26). AGES can also form cross-links within collagen fibers, which changes their structure and functionality. In combination with the abovementioned effects, this can result in damage to connective tissue in the joints, and eventually cause a condition called limited joint mobility (27).
3. Intracellular hyperglycemia also causes activation of *protein kinase C (PKC)*, which has several effects on gene expression within the cells (21). PKC can activate nuclear factor kappa B (NF-κB), which plays a pivotal role in the upregulation of inflammatory responses. For example, NF-κB regulates gene expression of IL-6, IL-1α, and TNF-α (28). Also, endothelin-1 (ET-1) is upregulated while endothelial nitric oxide synthase (eNOS) is downregulated, causing vasoconstriction and thereby abnormal blood flow (29, 30). Transforming growth factor beta (TGF-β) and plasminogen activator inhibitor-1 (PAI-1) are also upregulated, causing capillary and vascular occlusion (31, 32). An increased expression of vascular endothelial growth factor (VEGF) causes increased vascular permeability and angiogenesis (33). All these processes are harmful for the vasculature (21).
4. In the healthy situation, intracellular glucose is mainly metabolized through glycolysis. However, in a state of hyperglycemia, part of that glucose is diverted into another pathway known as the *hexosamine pathway*. This pathway comprises a complex cascade of reactions, leading to increased productions of cytokines (such as PAI-1, TGF-α, and TGF-β1) which in fact are harmful for the blood vessels (25).

Throughout this report, we will refer to these processes as *upstream* (ROS production) and *downstream* (polyol, AGEs and RAGE, PKC and hexosamine) pathways of hyperglycemia. Since endothelial cells are the major target cells of hyperglycemia, it is not surprising that highly vascularized organs are particularly susceptible to tissue damage. For example, damage to endothelial cells in the retina and kidney cause retinopathy (34, 35) and nephropathy (36, 37), respectively. Also, injury to the vulnerable vascular supply of the peripheral nervous system is likely to cause hypoxia in the endoneurium (38). On top of that, neuronal cells themselves are also susceptible for damage caused by hyperglycemia. This results in damage to the nerve, particularly in the most-distal fibers, eventually causing peripheral neuropathy (38, 39).

### Insulin Resistance

The second mechanism that is involved in chronic diabetic complications is insulin resistance. As its name already implies, insulin resistance is characterized by a reduced sensitivity of body cells to the actions of insulin. Since insulin resistance is closely related to obesity, it is more common in patients with T2DM, where it is an important cause for hyperglycemia. Besides its effect on blood glucose levels, insulin resistance also causes an excess flux of free fatty acids (FFAs) from adipose tissue into the bloodstream. This in turn increases the production and release of very low-density lipoprotein (VLDL) in the liver, resulting in a dyslipidemia (40). The role of dyslipidemia is discussed in the next section. However, the circulating FFAs caused by insulin resistance also play a direct role in the development of macrovascular complications (i.e., CVD). Increased flux of FFAs into arterial endothelial cells causes increased FFA oxidation in their mitochondria, eliciting ROS production and subsequently activating the same four pathways that we discussed in the previous section (polyol, AGEs, PKC, and hexosamine) (21, 41). Furthermore, protective enzymes such as eNOS and prostacyclin synthase are downregulated due to insulin resistance (42), and adhesion of leukocytes to endothelial cells is increased (43). Together, these processes result in enhanced vasoconstriction, inflammation, and procoagulant alterations. In the arterial endothelial cells, this favors the development of atherosclerotic lesions, which are the main cause of CVD (23, 44–46). A large prospective trial indeed showed that having insulin resistance almost doubled the risk for CVD (47).

In contrast to the macrovascular endothelial cells, FFA oxidation is not increased in microvascular endothelial cells in case of insulin resistance (21). However, insulin resistance might be involved in microvascular damage through other mechanisms. DM and obesity are both associated with selective impairment of some insulin signaling pathways (e.g., the PI3K pathway) that reduce nitric oxide availability and cause a compensatory hyperinsulinemia (48). The abundant insulin would then act through other signaling pathways that are less affected by DM or obesity (e.g., the Ras/MAPK pathway). The upregulation of these specific pathways, and the reduced availability of nitric oxide have unfavorable effects on the microvasculature, such as impaired angiogenesis and vasoreactivity (48).

### Dyslipidemia

Already announced in the insulin resistance section, dyslipidemia is the third pathogenic mechanism that plays a role in the development of diabetic complications. Dyslipidemia is more common in T2DM than in T1DM, and is typically characterized by high levels of triglycerides and small dense low density lipoprotein (sdLDL) cholesterol particles, in combination with low levels of high density lipoprotein (HDL) cholesterol (i.e., the “lipid triad”) (49). Typical characteristics of patients with T2DM, such as visceral adiposity, insulin resistance, and excess FFAs in the bloodstream play a major role in the development of this specific lipid profile. Because of its atherogenic properties, diabetic dyslipidemia is particularly important in the development of macrovascular complications (50). Several trials showed that lipid lowering therapy—especially with statins—indeed decreased the risk of CVD in patients with T2DM (51, 52). Dyslipidemia is also involved in microvascular complications, as shown in several intervention studies. Treatment with fibrates—hypolipidemic agents that lower triglyceride levels and increase HDL levels—prevented the progression of retinopathy (53), reduced albuminuria and prevented loss of glomerular filtration rate (GFR) (54). High levels of plasma triglycerides are also associated with progression of diabetic neuropathy (55). How dyslipidemia biologically contributes to the development of microvascular complications remains mostly unknown (56). However, it is suggested that oxidation of LDL cholesterol (oxLDL) causes increased ROS production in for example neuronal cells (57).

### Hypertension

Hypertension, the fourth mechanism contributing to diabetic complications, is more common in patients with DM compared to the general population (58). In fact, hypertension and T2DM in particular share many etiologic factors, such as obesity, insulin resistance, and inflammation (59). As we discussed before, patients with T2DM have a higher risk for CVD, mainly due to hyperglycemia, insulin resistance, and dyslipidemia. Having high blood pressure increases that risk even more (58). A large randomized controlled trial showed that tight blood pressure control in patients with T2DM significantly reduced the risk for CVD (60). Besides macrovascular complications, hypertension is also considered as an important risk factor for microvascular complications such as retinopathy (61) and nephropathy (36). In the previously mentioned study, tight blood pressure control also resulted in a significant reduction in the risk and progression of microvascular complications (60). The biologic contribution of hypertension to diabetic complications remains to be further elucidated. However, it was demonstrated that hypertension in patients with T2DM resulted in endothelial activation, reflected by increased levels of soluble adhesion molecules such as E-selectin and vascular cell adhesion molecule 1 (VCAM-1) (62). These adhesion molecules play in important role in the initiation of inflammation. Also, availability of nitric oxide might be decreased in the case of hypertension, resulting in vasoconstriction (63). Both processes are harmful for the vasculature and might eventually contribute to microvascular damage.

### Immune Dysfunction: Impaired Immune Response and Proinflammatory State

As we have shown in Figure 1, immune dysfunction plays a pivotal role in the pathogenesis of diabetic complications. DM can have a negative effect on several aspects of the immune system. For example, the innate (polymorphonuclear neutrophils [PMNs], macrophages, and monocytes) and adaptive (T-lymphocytes) immune responses are often impaired (64). More specifically, PMNs show impaired chemotactic, phagocytic, and microbicidal properties in patients with DM (65, 66). Also, adherence of microorganisms to several cell types, such as epithelial and endothelial cells, is increased (64). The impaired immune response is the major reason why patients with DM are more prone to opportunistic infections. Lower respiratory tract infections, urinary tract infections, bacterial and mycotic skin and mucous membrane infections are all found to be increased in patients with DM (67). However, the most impeding infection is probably the diabetic foot (68). Trauma, deformity, and peripheral neuropathy are the most common underlying causes of foot ulcerations in individuals with DM (69). In these patients, healing of ulcerations is impaired, with a crucial role for the so-called pathogenic triad: neuropathy, ischemia (due to vascular damage and endothelial dysfunction), and trauma (70). This healing abnormality, in combination with the impaired immune response, causes ulcers to become a *porte d'entrée* for opportunistic micro-organisms. As a result, the tissue can get infected, which has major implications for further development of the diabetic foot (71). Eventually, if the infection does not resolve, amputation is the only remaining solution to prevent sepsis (72, 73).

The immune dysfunction characterizing DM also manifests itself as a chronic pro-inflammatory state (11). As a response to the metabolic and hemodynamic disturbances discussed before, endothelial cells in the target tissues are activated. This is characterized by increased expression of adhesion molecules (e.g., intracellular adhesion molecule 1 [ICAM-1] and VCAM-1) and release of chemotactic factors (e.g., CCL5 and CCL5), which initiates immune cell recruitment and infiltration into the tissue. These immune cells, and other cells at sites of complications (e.g., adipocytes), release a wide variety of cytokines (e.g., IL-1β, IL-6, and TGF-α) that amplify the inflammatory response even further. If the inflammation remains unresolved and becomes chronic, it contributes to the development and progression of neuropathy (38), nephropathy (74), and retinopathy (75).

Finally, immune dysfunction itself can be one of the causes of DM as well, since we know that T1DM is a form of diabetes that is caused by autoimmune destruction of pancreatic β cell. These patients are also susceptible to develop other autoimmune diseases: Graves' disease, Hashimoto's thyroiditis, Addison's disease, vitiligo, celiac sprue, autoimmune hepatitis, myasthenia gravis, and pernicious anemia (1).

## Potential Oral Complications of Diabetes Mellitus

**Figure 2:**
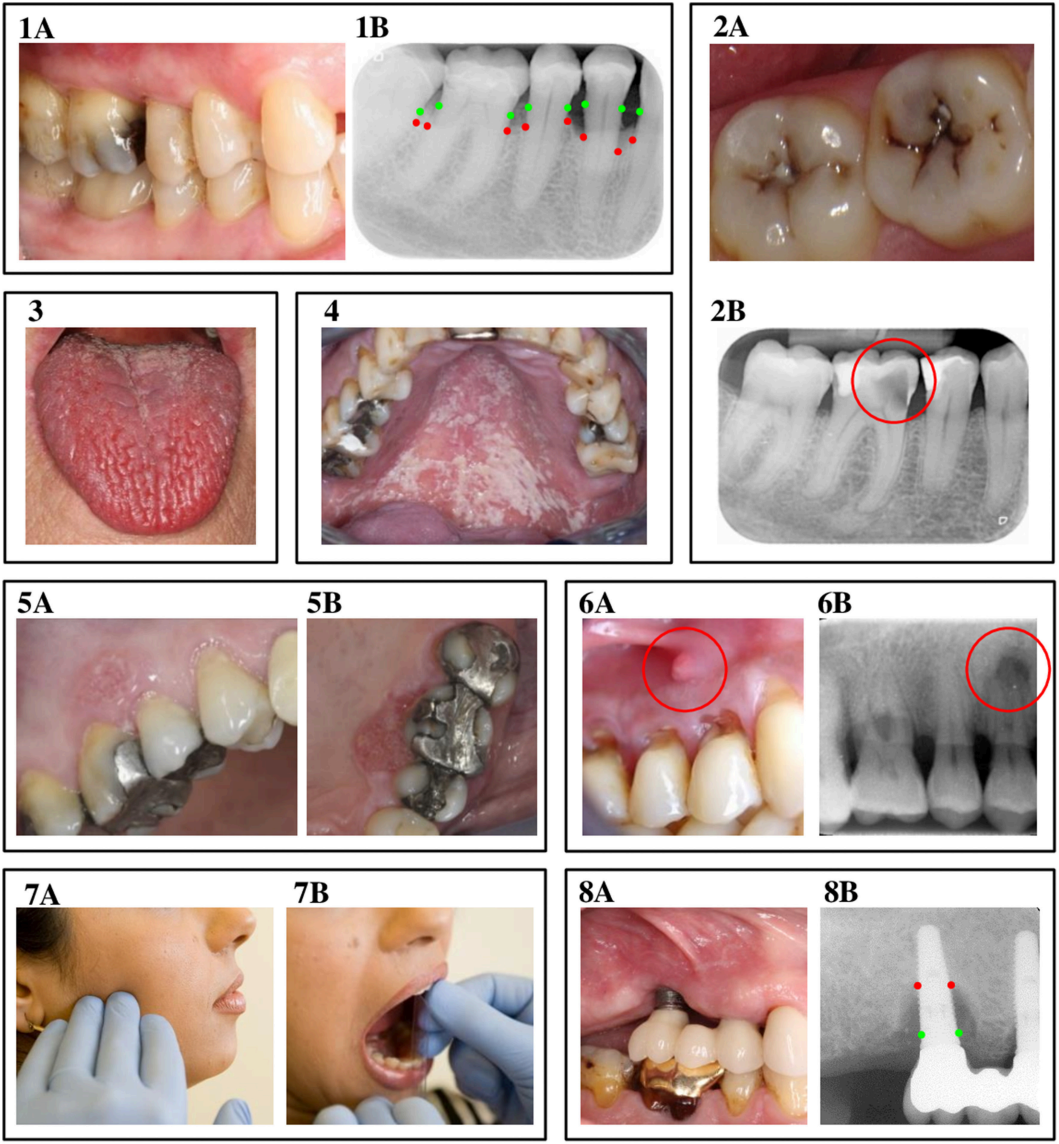
Oral complications of diabetes mellitus. *1A & 1B: Periodontitis*. A clinical view of periodontitis is shown in figure 1A, with the corresponding radiograph displayed in image 1B. The green dots in image 1B indicate where the bone level originally was, while the red dots show the actual bone level as a result of inflammation. *2A & 2B: Dental caries*. Image 2A displays a clinical view of dental caries, observed from the occlusal view. Picture 2B is a radiograph of an example of a deep carious lesion, marked by the red circle. *3. Hyposalivation*. The photo in image 3 shows a clinical view of fissured tongue, caused by severe hyposalivation. *4. Oral candidiasis*. Image 4 displays a clinical view of oral candidiasis, located at the palate. *5A & 5B: Oral cancer*. At image 5, a clinical representation of leukoplakia and oral cancer is shown at the buccal (5A) and palatal (5B) site. *6A & 6B: Apical periodontitis*. Figure 6A shows a fistula, caused by apical periodontitis, with image 6B as the corresponding radiograph, where the lesion at the apex of the tooth is marked by the red circle. *7A & 7B: Temporomandibular disorders*. Image 7A indicates the location of pain often seen in patients with temporomandibular disorders, while 7B shows a measurement of limited jaw opening, another symptom of temporomandibular disorders. *8A & 8B: Peri-implantitis*. At figure 8A, a clinical view of peri-implantitis is presented, with 8B as the corresponding radiograph. Again, the red dots indicate where ideally, the bone level should be, while the red dots show the actual reduced bone level as a result of the inflammatory process. The authors thank the following colleagues for providing clinical pictures and/or radiographs: Dr. AJP van Strijp (dental caries), Prof. Dr. M Laine (hyposalivation), Dr. P Wetselaar (temporomandibular disorders and apical periodontitis), all from ACTA, the Netherlands; Dr. RJJ van Es (oral cancer), UMC Utrecht, The Netherlands; Prof. Em. Dr. I van der Waal (oral candidiasis).

The previous section explained that chronic complications of DM are the result of persistent metabolic and hemodynamic disturbances that mainly target endothelial cells, typically affecting specific regions in the body. It has been proposed in literature that the oral cavity of patients with DM might be one of those regions, with an increased susceptibility for oral complications as a result (6, 76). This section aims to provide an up-to-date overview of the oral complications that are possibly associated with DM. Figure 2 presents clinical pictures and radiographs of the oral complications discussed in this review. Each oral complication is briefly introduced, after which we discuss the association with DM from an epidemiologic point of view. Next, we will use the five mechanisms we discussed before (*hyperglycemia, insulin resistance, dyslipidemia, hypertension*, and *immune dysfunction*) to assess possible pathogenic associations.

### Periodontal Diseases

#### Background

Periodontal disease can be subdivided into *gingivitis*, which is a reversible inflammation of the gum (gingiva) around the teeth without loss of support, and *periodontitis*, which has the clinical appearance of gingivitis but also shows irreversible destruction of the supporting structures around the teeth (root cementum, periodontal ligament, and alveolar bone). Approximately 30–50% suffer from any form of periodontitis, including the mild and moderate variant, while the prevalence of severe periodontitis in adults is estimated to be approximately 9–11% (77, 78). Severe periodontitis was ranked as the sixth most prevalent disease worldwide in 2010 (79–81). Several types of periodontitis have been described: aggressive or chronic periodontitis, necrotizing gingivitis and periodontitis and finally periodontal abscesses (82, 83). Recently, a new classification and case definition has been introduced. The proposed case definition extends beyond description based on severity to include characterization of biological features of the disease and represents a first step towards adoption of precision medicine concepts to the management of periodontitis (84). Periapical periodontitis—a specific type of periodontitis affecting the periodontium around the apex of the tooth—and peri-implantitis—a chronic inflammation affecting the tissues around dental implants—will be discussed in separate sections.

Periodontal disease is the result of an aberrant inflammatory host response to the biofilm that resides around the teeth. In some susceptible subjects, an initial gingivitis can progress into chronic periodontitis (82). Several risk factors that might influence the susceptibility for periodontitis have been established in literature, clustered in five categories: environment (the subgingival microbiome), genetics, systemic diseases (e.g., DM and HIV/AIDS), lifestyle (e.g., oral hygiene, smoking, diet), and tooth related factors (e.g., iatrogenic causes or occlusal problems) (85, 86). In chronic periodontitis, irreversible loss of supporting tissue surrounding the teeth (due to destruction of gingival connective tissue fibers, root cementum, periodontal ligament, and alveolar bone) results in deep periodontal pockets and attachment loss, which are commonly used to define a patient with periodontitis (87). This impairing process eventually causes loosening of the teeth and ultimately, when no periodontal treatment has been initiated, in tooth loss (88).

#### Relationship With Diabetes Mellitus: Epidemiology

Periodontal disease has been linked with DM for a long time. Since Loë suggested to consider periodontal disease as the sixth complication of DM in 1993 (89), periodontitis became the most researched oral complication of DM. As summarized in several narrative reviews (7, 8, 90–95), meta-analysis (96, 97), and a major consensus report (98), DM is considered as a risk factor for the development, progression, and severity of periodontitis.

Many cross-sectional studies show an increased prevalence of periodontitis in patients with DM. However, longitudinal studies are relatively scarce, even though these studies are necessary for establishing causal relationships. An important source of evidence is the research conducted with the Pima Indians in the 1990s, a population with one of the highest T2DM prevalence in the world. Indeed, diabetic subjects within this population displayed an increased prevalence of periodontal disease (99, 100). More importantly however, analysis of longitudinal data from this population also showed that the incidence of periodontitis in patients with (poorly controlled) diabetes was more than twice as high, compared to subjects with well-controlled or no DM (101). The risk for alveolar bone loss was higher in the poorly controlled diabetes group, as well as the severity of disease progression (102). Other prospective cohort studies confirmed the increased incidence of periodontitis in patients with (pre)diabetes (103, 104). Glycemic control appeared to be of particular importance, as patients with well-controlled diabetes showed a similar risk for periodontitis as matching individuals without DM (102, 105). This was confirmed in a large trial, where glycemic control rather than the etiology of DM was associated with deterioration of pocket depth and periodontal attachment loss (106). Improving glycemic control in patients with DM and periodontitis reduced inflammation, expressed as gingival bleeding (107). Solid evidence on the association between periodontal abscesses and DM is lacking. However, based on clinical experiences, it is speculated that—while a single periodontal abscess usually is the result of local factors—multiple recurring abscesses might indicate an underlying systemic cause, such as uncontrolled DM (83).

The majority of studies used the former classification of chronic periodontitis or at least did not specify the presentation of the disease. Since a new classification and case definition for periodontitis has recently been proposed, we decided not to distinguish different types of periodontitis in relation to DM in this review (84).

#### Relationship With Diabetes Mellitus: Pathogenesis

From an epidemiologic point of view, DM has an adverse effect on periodontal health. A limited number of longitudinal studies even indicate a possible causal relationship. However, the biologic mechanisms behind this relationship are still not completely understood. Several studies do show pathologic changes in the gingival vasculature of patients and animals with diabetes, compared to control subjects without DM. Examples are basement membrane thickening, angiogenesis, and an increase in osmotic tissue pressure (108–112). This strengthens the hypothesis that DM might affect periodontal tissue in a way similar to how it affects retinal, neural, and renal tissue, namely through vascular damage. We hope to elucidate this by separately discussing the same five pathological mechanisms (hyperglycemia, insulin resistance, dyslipidemia, hypertension, and immune dysfunction) that cause the well-known systemic complications of DM (Figure 1):

##### Hyperglycemia

As we discussed earlier, poor glycemic control increases the risk for periodontitis in patients with DM, and improvement in HbA_1c_ levels reduces periodontal inflammation (107). Apparently, just as in the well-known diabetic complications, hyperglycemia is a key pathological determinant for periodontitis. While the damaging processes through which hyperglycemia contributes to the development and progression of the well-known complications are well-established (21), this is not the case for DM-associated periodontal disease. However, there is evidence that the underlying upstream mechanism—oxidative stress—affects periodontal health as well (113). Low antioxidant levels are often interpreted as indicator for oxidative stress, and several studies indeed found decreased levels of antioxidants in serum, periodontal tissue, and saliva of patients with DM and periodontitis, compared to subjects without DM (114–117). One study found that the presence of T2DM in a rodent periodontitis model accelerated the decrease in gingival endothelial function (measured by Laser Doppler Flowmetry or LDF), mediated by oxidative stress (118).

Of the downstream effects of hyperglycemia (*polyol pathway, AGE/RAGE, PKC, and hexosamine pathway*) that might play a role in the pathogenesis of periodontal diseases, *AGEs*, and *RAGE* have been studied the most. We already mentioned that patients with DM are susceptible for joint related diseases, due to damage to the connective tissues in these joints, caused by AGEs accumulation. Periodontitis is also characterized by destruction of connective tissue, both in the gingiva and periodontal ligament, suggesting similar pathogenic pathways via AGE accumulation. AGEs and RAGE are both increased in gingival tissue (119) and saliva (120) of patients with T1DM and T2DM. The mechanisms by which AGEs are involved in periodontal tissue destruction are probably the same as in the well-known complications of DM: enhanced inflammation, impaired wound repair, and increased oxidative stress. In the specific case of periodontitis, this results in increased gingival connective tissue and periodontal ligament destruction and alveolar bone resorption (119–123). Increased levels of AGEs in serum of patients with DM were also significantly associated with deterioration of periodontitis (124). We know that AGEs are particularly important in the progression of periodontitis in persons with DM, because research showed that blocking RAGE reduced periodontal tissue breakdown (125).

There is limited evidence that the *polyol pathway* plays a role in periodontitis. Reduced glutathione (GSH) was one of the antioxidants that was found to be decreased in saliva (115, 117) and periodontal tissue (116) of patients with DM and periodontitis, compared to patients without DM. Another study also showed increased levels of oxidized glutathione (GSSH) in saliva (114). As mentioned before in this review, decreased levels of GSH and increased levels of GSSH are typical for increased polyol pathway flux caused by hyperglycemia. In another study, therapy with aldose reductase inhibitors—possibly obstructing flux of excess glucose into the polyol pathway—prevented alveolar bone loss in rats with DM. This could indicate that the polyol pathway is involved in the progression of periodontitis in patients with DM (126). However, a similar study showed similar results for non-diabetic rats, indicating that aldose reductase inhibitors might prevent alveolar bone loss through mechanisms other than obstructing the polyol pathway flux (127).

*Protein kinase C (PKC)* activity is also increased in patients with DM and periodontal disease (128), but whether it actually contributes to alveolar bone loss in these patients remains unclear. The possible role of the *hexosamine pathway* in hyperglycemia-associated periodontitis has not been studied yet.

##### Insulin resistance

Analysis of periodontal pocket depth in a large population without DM showed an independent association with our second pathogenic mechanism; insulin resistance. However, the association was interpreted as periodontal inflammation being a risk factor for insulin resistance, rather than the other way around (129). A comparable Korean cross-sectional study did not show such a relationship, but they did find impaired pancreatic β cell functioning in patients with periodontitis (130). In another large adult Korean population, insulin resistance was associated with periodontitis, but only in post-menopausal women (131). Furthermore, a large cohort from the United States suggested that the link between obesity and severe periodontitis was mediated through insulin resistance (132). However, the direction of the discovered relationships between insulin resistance and periodontitis could not be established in these cross-sectional studies. A study in Finland did not find an independent association between insulin resistance and periodontitis at baseline (133). However, in the same cohort, after 4 years of follow up, high levels of insulin resistance at baseline weakly but independently predicted the deepening of periodontal pockets (134). Also, in a non-diabetic rat model, obesity-induced insulin resistance resulted in endothelial dysfunction and inflammation of the gingiva, characterized by decreased eNOS expression and increased activity of PKC, NF-κB, and oxidative stress markers (135). Although this might indicate that insulin resistance negatively influences periodontal health, longitudinal studies with human subjects are necessary to confirm this hypothesis.

##### Dyslipidemia

The independent effect of lipid dysregulation on periodontal health has been studied in several populations without DM (136). Strikingly, the lipid profile typical for patients with DM (low levels of HDL; high levels of triglycerides and slightly increased LDL) was associated with worsened periodontal health, characterized by increased periodontal attachment loss, pocket depth, and bleeding gums (137). Comparable results were found in other cross-sectional studies, although it should be noted that different definitions of periodontitis and varying markers for dyslipidemia were used (138–140). The mechanism by which dyslipidemia contributes to periodontitis is not fully understood, and longitudinal studies to establish a causal relationship are missing. However, it is hypothesized that—similar to hyperglycemia—dyslipidemia stimulates a pro-inflammatory state, and thereby increases the susceptibility for periodontitis (141). Furthermore, it is thought that, besides glucose, lipids can also act as a source for ROS production in the gingiva through lipid peroxidation (LPO). Patients with DM and dyslipidemia have shown increased levels of markers for LPO (116, 142). These increased values were significantly correlated with parameters for periodontitis (% sites with bleeding, periodontal pockets ≥6 mm and pocket suppuration) and several inflammatory markers (IL-6, IL-10, TNF-α) (142). A relatively new insight is the beneficial effect of statin therapy on periodontal treatment outcome in patients without DM, as described in several recent systematic reviews (143–145). However, it should be noted that this is not necessarily caused by statins' lipid lowering effect; it could be the result of pleiotropic features of statins, such as antioxidant and anti-inflammatory effects (143).

##### Hypertension

It is hypothesized that hypertension is associated with periodontal disease, although the majority of studies interpret this relationship as periodontitis being a risk factor for hypertension through inflammation (146). This hypothesis is mainly based on findings that periodontal therapy is beneficial for systolic and diastolic blood pressure (147). However, longitudinal research in the opposite direction is lacking; hypertension might very well be a risk factor for developing periodontitis as it is for other diabetic complications (148, 149). A recent systematic review and meta-analysis found an OR of 1.50 (95% CI: 1.27–1.78) for the association between the presence of hypertension and periodontitis, but again, the direction could not be established (150). It should also be stated that there is a major overlap in risk factors for both conditions. However, a few animal studies show that hypertension might negatively affect alveolar bone quality (151) and gingival vasculature (152), and that it can exacerbate experimental periodontitis (153). Treatment with an anti-hypertensive drug in an animal model with periodontitis resulted in decreased levels of inflammatory cytokines (IL-1β and TNF-α), reduced expression of markers for alveolar bone breakdown (MMP-2, MMP-9, RANKL/RANK), increased expression of a bone growth promoting marker (osteoprotegerin or OPG) and finally reduced alveolar bone loss (154).

##### Immune dysfunction: impaired immune response and proinflammatory state

As we stated in the background section, periodontitis is a chronic disease characterized by an aberrant inflammatory host response to the biofilm. However, in patients with DM, this pro-inflammatory state seems to be even more exaggerated. PMNs, but also monocytes and macrophages, produce more inflammatory mediators and ROS, which could contribute to tissue destruction in periodontal disease (128, 155–158). Prolonged retention of the PMN infiltrate ensures that the potentially damaging inflammatory response is maintained longer in patients with DM (159–161). Also, patients with DM show increased levels of inflammatory cytokines in gingival crevicular fluid and gingival tissue, such as IL-1β and IL-6, compared to healthy subjects (157, 162–166). TNF-α also contributes to periodontal tissue destruction by enhancing the inflammatory response and activating osteoclasts responsible for bone resorption (167). Another interesting cytokine is receptor activator of NF-κB ligand (RANKL), since it is also involved in the inflammatory processes seen in other diabetic vascular complications (44). In the case of periodontal disease, RANKL and the ratio with OPG determine whether bone is generated (low ratio) or resorbed (high ratio). Especially in patients with poorly controlled diabetes, this ratio is increased in the periodontal tissue (168–170) and gingival crevicular fluid (171, 172), with alveolar bone resorption as a consequence. Treatment with OPG, and thereby decreasing the RANKL/OPG ratio, even reversed alveolar bone loss in diabetic mice. This suggests that this ratio is important for the development or prevention of periodontitis in the presence of DM (169).

We discussed before that patients with DM are susceptible for opportunistic infections because of an impaired innate and adaptive immune response. In the case of periodontitis, especially the PMN response seems to be impaired, characterized by reduced chemotaxis (173, 174). This might cause opportunistic pathogens in the biofilm to persist over time, thereby causing the aberrant inflammation to retain, which enhances the progression of periodontitis (173–175). In that perspective, (severe) periodontitis in patients with DM can best be compared to the diabetic foot. Just as in this condition, the exaggerated inflammatory response to the unresolved infection with opportunistic micro-organisms is retained, which—in combination with poor wound healing—sometimes even results in tooth loss (176, 177).

#### Concluding Comments

Table 1 summarizes the studies on which the section above was based. Both prevalence and incidence of periodontitis are increased in patients with DM. There are many similarities to the pathogenesis of the well-known complications of DM. Glycemic control seems to be particularly important in the development and severity of periodontitis. However, there are indications that other metabolic disturbances also play a role, such as dyslipidemia, insulin resistance, and especially immune dysfunction.

**Table 1:** Overview of studies investigating the association between diabetes mellitus and periodontal disease.

**Study**	**Study population**	**Study design**	**Relationship?**	**Authors' conclusions**
**EPIDEMIOLOGY**				
Chavarry et al. (97)	49 cross-sectional studies (17 on T1DM [*n* = 1,678], 26 on T2DM [*n* = 13,773] and 6 on both types [*n* = 17,427]). 8 longitudinal studies (total *n* = 644).	Systematic review with meta-analysis	*Yes*	T2DM increased the risk for development and progression of periodontal disease. The meta-analysis revealed statistically significant mean differences in pocket depth (0.46, 95% CI: 0.01–0.91) and clinical attachment loss (1.00, 95% CI: 0.15–1.84) when comparing patients with T2DM and healthy controls. The positive mean differences indicated more periodontitis. This could not be established for patients with T1DM.
Chiu et al. (103)	Group 1: Patients without DM (FPG < 100 mg/dL) (*n* = 4,033). Group 2: Patients with pre-diabetes (FPG 100–125 mg/dL) (*n* = 297). Group 3: Patients with T2DM (medical history or FPG ≥126 mg/dL) (*n* = 57). All groups were periodontally healthy at baseline.	Prospective cohort study	*Yes*	After correcting for all possible confounding factors, patients with (pre-diabetes had a higher incidence of periodontal disease (community periodontal index [CPI] of 3 [a 4–5 mm pocket] or 4 [a pocket ≥6 mm]) compared to subjects with normal FPG levels at baseline.
Demmer et al. (106)	Group 1: Diabetes free subjects (*n* = 2,280). Group 2: Incident patients with T2DM (*n* = 79). Group 3: Patients with controlled T2DM (HbA_1c_ 6.5–7.0%) (*n* = 80). Group 4: Patients with uncontrolled T2DM (HbA_1c_ >7.0%) (*n* = 72). Group 5: Patients with controlled T1DM (HbA_1c_ 6.5-7.0%) (*n* = 43). Group 6: Patients with uncontrolled T1DM (HbA_1c_ >7.0%) (n = 72).	Prospective cohort study	*Yes*	Patients with uncontrolled T2DM had a significant higher increase in pocket depth over 5 years, compared to the healthy subjects. Patients with either uncontrolled T1DM or T2DM had significant higher increase in attachment loss over 5 years, compared to diabetes free subjects.
Jimenez et al. (104)	Group 1: Patients with T2DM (self-reported; *n* = 2,285; mean age = 54.7 ± 8.9 years). Group 2: Patients without DM (*n* = 32,962; mean age = 53.4 ± 9.6 years).	Prospective cohort study	*Yes*	Patients with T2DM had an adjusted 29% greater risk of developing periodontitis, compared to patients without DM (HR = 1.29; 95% CI:1.13–1.47).
Löe (89)	Group 1: Pima Indians with T2DM (*n* = 693; 261 males, 432 females). Group 2: Pima Indians without DM (*n* = 1,487; 644 males, 843 females).	Retrospective cohort study	*Yes*	Both prevalence and incidence were increased in patients with DM, compared to the control subjects. Furthermore, in the subjects with DM, a higher prevalence of edentulism was found, which also increased with a longer duration of the diabetic conditions.
Nelson et al. (101)	Group 1: Pima Indians with T2DM (*n* = 720). Group 2: Pima Indians without DM (*n* = 1,553).	Cohort study	*Yes*	Both incidence and prevalence of periodontal disease was higher in patients with DM, compared to subjects without DM, after correction for age and gender. They conclude that periodontal disease should be considered as a non-specific complication of T2DM.
Taylor et al. (102)	Group 1: Patients with poorly controlled T2DM (HbA_1c_ ≥9.0%) (*n* = 7; 4 males, 3 females; median age = 26 years). Group 2: Patients with better controlled T2DM (HbA_1c_ < 9.0%) (*n* = 14; 3 males, 11 females; median age = 27 years). Group 3: Controls without DM (*n* = 338; 138 males, 200 females; median age = 21 years).	Retrospective cohort study	*Yes*	Patients with poorly controlled DM had a greater risk for alveolar bone loss progression. Also, this progression was more severe in patients, compared to controls.
**PATHOGENESIS**				
**Hyperglycemia**				
*Polyol pathway*				
Kador et al. (126)	Group 1: Control rats (*n* = 5). Group 2: Diabetic rats (*n* = 8). Group 3: Diabetic rats, treated with aldose reductase inhibitor (ARI) (*n* = 8). Rats used: Sprague-Dawley rats (male, diabetes induced by Streptozotocin injection, experimental periodontitis induced by injection of LPS from *Escherichia coli*).	Longitudinal animal study	*Yes*	Injection of LPS resulted in significant bone loss in the control group and untreated diabetic rats. However, in the group treated with ARI, this was not observed. This might indicate that by obstructing a crucial step in the polyol pathway, alveolar bone loss in a diabetic rat model was prevented.
Kador et al. (127)	Group 1: Diabetic rats (*n* = 30; 8 on standard diet, 22 on ARI diet). Group 2: Non-diabetic rats (*n* = 30, 8 on standard diet, 22 on ARI diet). Rats used: Sprague-Dawley rats (male, diabetes induced by Streptozotocin injection, experimental periodontitis induced by injection of LPS from *Porphyromonas Gingivalis)*.	Longitudinal animal study	*Unclear*	Aldose reductase inhibitors indeed prevented alveolar bone loss in diabetic rats, but in control rats as well.
*Advanced glycation endproducts*				
Chang et al. (122)	Group 1: T1DM rats (*n* = 18). Group 2: Control rats (*n* = 18). Rats used: Sprague-Dawley rats (male, diabetes induced by Streptozotocin injection, a tooth-associated osseous periodontal defect was surgically created).	Longitudinal animal study	*Yes*	Healing of bony defects was significantly lower in diabetic rats, compared to healthy rats. The AGE-RAGE interaction was enhanced by both inflammatory status and diabetic conditions, indicating a role in the impaired periodontal wound healing seen in patients with DM.
Lalla et al. (125)	Group 1: T1DM mice (*n* = 77; C57BL/6; subdivided into different groups by treatment dose). Group 2: Non-diabetic control mice (*n* = 12).	Longitudinal animal study	*Yes*	Blockade of RAGE reduced alveolar bone loss and lowered levels of inflammatory cytokines such as TNF-α and IL-6. This indicates a link between levels of RAGE and periodontal destruction in patients with DM.
Schmidt et al. (123)	*Animal* Group 1: Diabetic mice (CD_1_) Group 2: Non-diabetic control mice. *Human* Group 1: Patients with T1DM (*n* = 1) or T2DM (*n* = 3) and periodontitis Group 2: Patients without DM, with periodontitis (*n* = 5).	Cross-sectional human and animal study	*Yes*	AGEs appeared to accumulate in the gingival tissue of both mice and humans with DM. In parallel with this, enhanced oxidative stress was observed in patients with DM, compared to controls without DM, linking AGE accumulation to periodontal destruction.
Takeda et al. (124)	Group 1: Patients with T2DM and periodontitis (*n* = 69). Group 2: Patients with T2DM without periodontitis (*n* = 28).	Cross-sectional study	*Yes*	AGEs were the only biomarker in serum that showed a significant relationship with periodontal disease.
Yoon et al. (120)	Group 1: Patients with DM (*n* = 52; 26 males, 26 females; mean age = 57 ± 13 year). Group 2: Age-matched controls without DM (*n* = 47; 17 males, 30 females; mean age = 41 ± 14 years).	Cross-sectional study	*Yes*	Significantly more AGEs were found in saliva of patients with DM, indicating involvement in the pathogenesis of periodontal disease as a complication of DM.
Zizzi et al. (119)	Group 1: Periodontally healthy subjects without DM (*n* = 16). Group 2: Patients with periodontitis, without DM (*n* = 16). Group 3: Patients with T1DM and periodontitis (n = 16). Group 4: Patients with T2DM and periodontitis (n = 16).	Cross-sectional study	*Yes*	In patients with T1DM or T2DM and periodontal disease, gingival AGEs are increased. In this study, duration of DM could be a determining factor for the accumulation of AGEs.
*Protein kinase C*				
Karima et al. (128)	Group 1: Patients with DM (*n* = 50; 12 T1DM, 38 T2DM; 30 males, 20 females; mean age = 50.9 years) Group 2: Healthy subjects without DM (*n* = 45, 30 males and 15 females; mean age = 48.3 years).	Cross-sectional study	*Yes*	Neutrophils of patients with DM produced significantly higher levels of superoxide and displayed higher protein kinase C activity compared to those of healthy controls. This indicates a more pro-inflammatory state. Furthermore, there was an association between metabolic control and severity of periodontal disease in patients with DM.
**Insulin resistance**				
Demmer et al. (129)	Patients without DM (*n* = 3,616; 50% male, 50% female; mean age = 41 ± 0.4 years).	Cross-sectional study	*Unclear*	An association between periodontal pocket depth and insulin resistance was found. However, the cross-sectional design leaves the association open for interpretation. Also, in the regression analysis, insulin resistance was considered as outcome variable, and pocket depth as risk factor.
Genco et al. (132)	Group 1: Patients with obesity (BMI ≥27 kg/m^2^), without DM (*n* = 5,326; 45.0% male; mean age = 45.2 ± 0.2 years). Group 2: Patients without obesity and DM (*n* = 7,041; 48.3% male; mean age = 42.2 ± 0.2 years).	Cross-sectional study	*Yes*	Multiple regression analysis showed that obese patients with high levels of insulin resistance were more likely to suffer from severe periodontitis, compared to obese patients with low levels insulin resistance.
Islam et al. (130)	Group 1: Patients with periodontitis (*n* = 5,992; 52.9% male; mean age = 55.4 ± 13.3 years) Group 2: Patients without periodontitis (*n* = 13,130; 38.7% male; mean age = 46.3 ± 16.4 years).	Cross-sectional study	*No*	There were no differences in insulin resistance between patients with or without periodontitis. However, patients with periodontitis had decreased pancreatic β cell functioning, compared to patients without periodontitis.
Lim et al. (131)	Group 1: Men with periodontitis (*n =* 2,055; mean age = 47.2 ± 0.4 years). Group 2: Men without periodontitis (*n =* 5,005; mean age = 41.3 ± 0.4 years). Group 3: Pre-menopausal women with periodontitis (*n =* 641; mean age = 40.1 ± 0.4 years). Group 4: Pre-menopausal women without periodontitis (*n =* 3,801; mean age = 34.8 ± 0.2 years). Group 5: Post-menopausal women with periodontitis (*n =* 763; mean age = 59.4 ± 0.4 years). Group 6: Post-menopausal women without periodontitis (*n =* 2,487; mean age = 62.6 ± 0.3 years).	Cross-sectional study	*Yes*	An association was found only in post-menopausal women, who showed a higher periodontitis prevalence when their insulin resistance increased. This was not found for pre-menopausal women or men.
Mizutani et al. (135)	Group 1: Male Zucker Fatty rats (ZF-*fa/fa*) (*n =* 12). Group 2: Lean matched controls (ZL-*fa/+*) (*n =* 12).	Cross-sectional animal study	*Yes*	The obesity induced insulin resistance resulted in increased oxidative stress, activation of PKC, decreased gingival endothelial functioning and increased inflammation, possibly contributing to the progression of periodontitis.
Timonen et al. (134)	Adult subjects without diabetes (*n =* 157), divided into three tertiles of insulin resistance: Group 1: Lowest tertile (*n =* 58; 20.7% male; mean age = 41.2 ± 8.3 years). Group 2: Intermediate tertile (*n =* 55; 34.6% male; mean age = 44.7 ± 9.5 years). Group 3: Highest tertile (*n =* 44; 25.0% male; mean age = 44.2 ± 10.7 years).	Prospective cohort study	*Yes*	Insulin resistance predicted the formation of periodontal pockets (4 mm or deeper) over a period of 4 year (IRR = 1.7, 95% CI: 1.1–2.7) after adjusting for confounders.
Timonen et al. (133)	Adult subjects without diabetes (*n =* 2,050), divided into five quintiles, ranging from lowest to highest insulin resistance levels.	Cross-sectional study	*No*	There was a crude association between insulin resistance and periodontal infection (measured by number of teeth with deepened pockets). However, when corrected for BMI, this association disappeared.
**Dyslipidemia**				
Awartani and Atassi (138)	Group 1: Otherwise systemically healthy female subjects with hyperlipidemia (*n =* 30; mean age = 47.1 ± 5.0 years). Group 2: Systemically healthy female subjects (*n =* 30; mean age = 46.3 ± 4.4 years).	Cross-sectional study	*Yes*	Mean probing pocket depth and attachment loss was significantly higher in the hyperlipidemic group, who also had a higher bleeding on probing index.
Bastos et al. (142)	Group 1: Patients with poorly controlled T2DM and dyslipidemia (*n =* 30; 12 males, 18 females; mean age = 48.0 ± 7.6 years) Group 2: Patients with well-controlled T2DM and dyslipidemia (*n =* 30; 10 males, 20 females; mean age = 50.3 ± 6.7 years) Group 3: Patients without T2DM, with dyslipidemia (*n =* 30; 13 males, 17 females; mean age = 49.0 ± 7.5 years) Group 4: Patients without T2DM and dyslipidemia (*n =* 30; 11 males, 19 females; mean age = 45.9 ± 5.9 years)	Cross-sectional study	*Yes*	Markers for lipid peroxidation were significantly increased in patients with T2DM. These increased values significantly correlated with periodontal parameters (% sites with bleeding, pocket depth ≥6 mm and pocket suppuration) and several inflammatory markers (IL-6, IL-10, TNF-α).
Fentoglu et al. (137)	Group 1: Subjects with hyperlipidemia (*n =* 51; 16 males, 35 females; mean age = 49.4 ± 6.0 years). Group 2: Normolipidemic subjects (*n =* 47; 27 males, 20 females; mean age = 47.3 ± 8.1 years).	Cross-sectional study	*Yes*	The hyperlipidemic group showed higher values of periodontal parameters (mean probing pocket depth, clinical attachment loss, bleeding on probing and plaque index).
Lee et al. (140)	Adult subjects (*n =* 15,534; 49.8% males; mean age = 44.9 years).	Cross-sectional study	*Yes*	Multivariate logistic regression analyses showed an association between dyslipidemia (hyper TC, hyper TG and hypo HDL-c) and periodontitis, after correction for demographic and general health characteristics (including DM).
Sangwan et al. (139)	Group 1: Normolipidemic subjects (*n =* 46; 22 males, 24 females; mean age = 42.5 ± 9.9 years). Group 2: Hyperlipidemic patients with statin treatment (*n =* 50; 29 males, 21 females; mean age = 45.6 ± 9.9 years). Group 3: Hyperlipidemic patients without statin treatment (*n =* 44; 25 males, 19 females; mean age = 41.3 ± 10.0 years).	Cross-sectional study	*Yes*	Hyperlipidemic patients without statin treatment had increased probing pocket depth and gingival index, compared to normolipidemic subjects and hyperlipidemic patients with statin treatment. Pocket depth was associated with TC, TG and LDL-c, while clinical attachment loss was associated with TC and LDL-c.
**Hypertension**				
Araujo et al. (154)	Group 1: Healthy Wistar albino rats (*n =* 10) Group 2: Ligated Wistar rats, fed with water (*n =* 10) Group 3: Ligated Wister rats, treated with 1 mg/kg telmisartan (TELM) (*n =* 10) Group 4: Ligated Wister rats, treated with 5 mg/kg TELM (*n =* 10) Group 5: Ligated Wister rats, treated with 10 mg/kg TELM (*n =* 10)	Longitudinal animal study	*Yes*	Anti-hypertensive treatment with TELM, especially the 10 mg/kg dose, resulted in decreased levels of inflammatory markers (IL-1β, TNF-α), reduced expression of markers for bone loss (MMP-2, MMP-9, RANKL/RANK, OPG), and actually reduced alveolar bone loss. It should be noted that normotensive rats were used in this study.
Bastos et al. (151)	Group 1: Normotensive Wistar rats (*n =* 15) Group 2: Spontaneously hypertensive rats (SHR), treated (*n =* 15) Group 3: Spontaneously hypertensive rats, not treated (*n =* 15)	Longitudinal animal study	*Yes*	In both treated and untreated hypertensive rats, increased bone loss and decreased bone density were observed, compared to the normotensive rats.
Castelli et al. (152)	Group 1: Normotensive control rats (*n =* 5). Group 2: Renovascular hypertensive rats (*n =* 20, 9 lost to follow up). Rats used: Sprague Dawley (male, adult).	Longitudinal animal study	*Unclear*	Morphologic changes to the gingival blood vessels were observed in the hypertensive rats, but not in the control rats. However, alveolar arterioles and pulp tissues were not affected.
Leite et al. (153)	Group 1: Normotensive Wistar rats (*n =* 6) Group 2: Spontaneously hypertensive rats (*n =* 6). In both groups, each rat had a site with ligature-induced periodontitis and a control site.	Longitudinal animal study	*Yes*	In the hypertensive rats, all ligated sites – where periodontitis was induced – showed moderate to severe alveolar damage. In the normotensive rats, only mild or even no damage was observed at the ligated sites. Hypertension possibly aggravates tissue damage in periodontitis.
**Immune dysfunction**				
Duarte et al. (163)	Group 1: Periodontally healthy subjects without DM (*n =* 10; 40% male, 60% female; mean age = 39.57 ± 2.85). Group 2: Patients without DM, with moderate- to-severe chronic periodontitis (*n =* 20; 33% male; 67% female; mean age = 37.50 ± 4.11). Group 3: Patients with T2DM and periodontitis (*n =* 20; 40% male; 60% female; mean age = 40.86 ± 3.37).	Cross-sectional study	*Yes*	Levels of IL-1β and IL-6 in periodontally inflamed tissue of patients with DM were significantly higher than those in the control group.
Duarte et al. (170)	Group 1: Periodontally healthy subjects without DM (*n =* 10; 40% male, 60% female; mean age = 39.57 ± 2.85). Group 2: Patients without DM, with moderate- to-severe chronic periodontitis (*n =* 20; 33% male; 67% female; mean age = 37.50 ± 4.11). Group 3: Patients with T2DM and periodontitis (*n =* 20; 40% male; 60% female; mean age = 40.86 ± 3.37).	Cross-sectional study	*Yes*	Lower levels of IL-10 (an anti-inflammatory cytokine) and OPG (an anti-resorption molecule) are observed in patients with DM. Together with the increased levels of their antagonists (IL-6 and RANKL, respectively), the balance between bone resorption and formation is negatively influenced by the diabetic state, leading to a greater periodontal breakdown.
Engebretson et al. (162)	Patients with T2DM (*n =* 45; 45% male, 55% female; mean age = 54.0 ± 9.8 years).	Cross-sectional study	*Yes*	Higher levels of HbA_1c_ were associated with higher levels of the pro-inflammatory cytokine IL-1β in GCF. This was on its turn correlated with worse clinical periodontal measures (pocket depth, clinical attachment loss, bleeding).
Engebretson et al. (175)	Group 1: Patients with T2DM (*n =* 45; 45% female, 55% male; mean age = 54.2 years). Group 2: Control group without DM (*n =* 32; 47% female; mean age = 42.0 years).	Cross-sectional study	*Yes*	Patients with DM showed an insufficient PMN response in the crevice (measured in GCF), which may (partly) contribute to the development and severity of periodontal disease.
Gyurko et al. (155)	Group 1: Chronic hyperglycemia mice (Akita; *n =* 28). Group 2: Age- and gender matched control mice (wild-type C57BL/6; *n =* 28).	Longitudinal animal study	*Yes*	Mice with hyperglycemia had an exaggerated inflammatory response as their leukocytes were primed for marginalization and superoxide production. However, transmigration was impaired. This could contribute to periodontal tissue damage by a weakened immune response to periodontal pathogens as well as by increased local production of free radicals.
Kardeşler et al. (165)	Group 1: Patients with periodontitis and T2DM (*n =* 17; 5 males, 12 females; mean age = 47.35 ± 8.3) Group 2: Patients with periodontitis, without DM (*n =* 17; 9 males, 8 females; mean age = 49.12 ± 6.6) Group 3: Systemically & periodontally healthy controls (*n =* 17; 3 males, 14 females; mean age = 40.65 ± 6.7)	Cross-sectional study	*Unclear*	The researchers did find increased levels of certain cytokines associated with inflammation in the GCF of patients with DM, compared to the healthy control subjects. However, there were no differences between group 1 (patients with DM and periodontitis) and group 2 (systemically healthy patients with periodontitis).
Kardeşler et al. (164)	Group 1: Patients with periodontitis and T2DM (*n =* 20; 13 males, 7 females; mean age = 53.6 ± 6.0 years). Group 2: Patients with periodontitis, without DM (*n =* 22; 10 males, 12 females; mean age = 49.6 ± 8.2 years).	Cohort study	*Yes*	After initial periodontal treatment, both groups showed clinical improvement. However, after 1 month the situation in the group with DM worsened, reflected by an increase in cytokines associated with increased inflammation, measured in the GCF (IL-6, tPA, and PAI-2).
Lappin et al. (168)	Patients with T1DM (*n =* 63; 30 males, 33 females), divided as: Group 1: “Low HbA_1c_” (*n =* 30; mean age = 34 years). Group 2: “High HbA_1c_” (*n =* 33; mean age = 40 years). Group 3: Controls without DM (*n =* 38; 16 males, 22 females; mean age = 40 years).	Cross-sectional study	*Yes*	Patients with DM displayed lower RANKL:OPG ratio, which would suggest that they are not susceptible for increased bone resorption via that specific pathway. However, lower levels of osteocalcin (a marker for bone formation) were also observed in patients with DM. It is suggested that this causes an insufficient repair response following bone loss, explaining the susceptibility for periodontal disease progression often seen in patients with DM.
Liu et al. (160)	Group 1: Diabetic rats (type-2 Zucker diabetic fatty (ZDF)). Group 2: Normoglycemic control rats. Periodontitis was induced by tying silk ligatures soaked with *Porphyromonas gingivalis* around the maxillary second molars or mandibular first molars,	Longitudinal animal study	*Yes*	There was a significant difference in retention of the inflammatory infiltrate (PMNs and mononuclear cells) between diabetic and non-diabetic rats after inducing experimental periodontitis. Consequently, a greater periodontal destruction was found in diabetic rats.
Mahamed et al. (169)	Group 1: Diabetic mice (T1DM model, NOD/LtJ). Group 2: Normoglycemic mice. Periodontitis was induced by oral inoculation with *Actinobacillus Actinomycetemcomitans*.	Longitudinal animal study	*Yes*	Diabetic mice presented higher alveolar bone breakdown compared to normoglycemic mice. The expression of RANKL by t-cells seemed to play an important role, and treatment with OPG prevented alveolar bone loss.
Manouchehr-Pour et al. (173)	Group 1: Patients with T1DM and severe periodontitis (*n =* 8). Group 2: Patients with T1DM and mild periodontal disease (*n =* 6).	Cross-sectional study	*Yes*	Patients with DM suffering from severe periodontitis exhibited a significant decrease in neutrophil chemotaxis, compared to patients with DM and mild periodontal
	Group 3: Patients without DM, with severe periodontitis (*n =* 7). Group 4: Patients without DM, with mild periodontal disease (*n =* 11).			disease and patients without DM. Impaired PMN chemotaxis might contribute to the severity of periodontitis in patients with DM.
McMullen et al. (174)	Group 1: Patients with a family history of diabetes, without overt DM themselves and with severe generalized alveolar bone loss (*n =* 24; 10 males, 14 females; mean age = 35.9 years). Group 2: Patients diagnosed with adult periodontitis, without family history of diabetes (*n =* 20; 10 males, 10 females; mean age = 41.8 years). Group 3: Age- and gender matched healthy control subjects.	Cross-sectional study	*Yes*	Patients with a family history of DM had impaired PMN chemotaxis, compared to patients without a family history of DM. They suggest that this impaired PMN function or number predisposes a diabetes patient to develop more severe periodontal disease.
Naguib et al. (161)	Group 1: Diabetic mice (*Db/db, n =* 6). Group 2: Normoglycemic mice (*Db/+*).	Longitudinal animal study	*Yes*	Diabetic mice that were inoculated with a periodontal pathogen (*Porphyromonas gingivalis*) presented a prolonged infiltration of PMNs in the inflamed tissue, compared to the non-diabetic mice.
Ross et al. (166)	Group 1: Patients without DM and periodontitis (*n =* 8; 1 male, 7 females; mean age = 59 ± 15 years). Group 2: Patients without diabetes, with periodontitis (*n =* 17; 11 males, 6 females; mean age = 50 ± 13 years). Group 3: Patients with DM and periodontitis (*n =* 10; 6 males, 4 females; mean age = 57 ± 12 years).	Cross-sectional study	*Yes*	There were significant differences in periodontal expression of IL-6 between all groups. Patients with both periodontal disease and DM had the highest levels, the controls showed the lowest expression.
Salvi et al. (158)	Group 1: Patients with T1DM (*n =* 32; aged 22-81 years). Group 2: Control subjects without DM (*n =* 17; aged 30-75 years).	Cross-sectional study	*Yes*	Patients with DM showed a significant higher monocytic TNF-α secretion in the presence of a periodontal pathogen (*Porphyromonas gingivalis*), which is associated with an increased periodontal disease severity.
Salvi et al. (157)	Group 1: Patients with T1DM (*n =* 39; aged 22-81). Group 2: Healthy control subjects (*n =* 64; aged 20-73).	Cross-sectional study	*Yes*	Concentrations of prostaglandin E_2_ (PGE_2_) and IL-1β in the GCF of patients with DM was significantly higher compared to the concentrations in subjects without DM. Furthermore, as a response to exposure to periodontal pathogens, monocytes in diabetic produced more PGE_2_ and IL-1β than those in controls.
Santos et al. (172)	Group 1: Patients with well-controlled T2DM (*n =* 18; 8 males, 10 females; mean age = 51.2 ± 9.9 years). Group 2: Patients with poorly controlled T2DM (*n =* 20; 9 males, 11 females; mean age = 53.4 ± 8.0 years).	Quasi-experiment	*Yes*	Overall, RANKL/OPG ratios were higher in patients with poorly controlled DM. Furthermore, after initial treatment, these ratios decreased in the well-controlled patients, but not in the poorly controlled. Therefore, metabolic control seems to play a role in periodontal bone resorption.
Sima et al. (156)	Group 1: Diabetic mice (Akita). Group 2: Healthy mice (wild-type C57BL/6).	Cross-sectional animal Study	*Yes*	The elevated blood glucose levels in diabetic mice resulted in increased leukocyte marginalization and macromolecule extravasation in the gingival vasculature. This represented a pro-inflammatory state, which might contribute to periodontal disease.
Vieira Ribeiro et al. (171)	Group 1: Patients with T2DM and chronic periodontitis (*n =* 37; 16 males; 21 females; mean age = 52.5 ± 8.7). Group 2: Systemically healthy subjects with chronic periodontitis (*n =* 20; 8 males, 12 females; mean age = 51.5 ± 8.3 years).	Cross-sectional study	*Yes*	Higher levels of OPG, sRANKL, IFN-γ, IL-17, and IL-23 (pro-inflammatory) and lower levels of IL-4 (anti-inflammatory) were observed in the GCF of patients with DM, compared to control subjects.

*T1DM, Diabetes Mellitus Type 1; T2DM, Diabetes Mellitus Type 2; FPG, Fasting Plasma Glucose; LPS, Lipopolysaccharide; ARI, Aldose Reductase Inhibitor; (R)AGE, (Receptor for) Advanced Glycation End Products; TNF-α, Tumor Necrosis Factor Alpha; IL, Interleukin (e.g., IL-1β or IL-6); PKC, Protein Kinase C; IRR, Incidence Rate Ratio; TC, Total Cholesterol; TG, Triglycerides; LDL-c, Low-density Lipoprotein Cholesterol; HDL-c, High-density Lipoprotein Cholesterol; TELM, Telmisartan; MMP, Matrix Metalloproteinase; (s)RANK(L), (soluble) Receptor Activator of Nuclear factor Kappa B (Ligand); OPG, Osteoprotegerin; GCF, Gingival Crevicular Fluid; PMN, Polymorphonuclear Neutrophil; tPA, Tissue Plasminogen Activator; PAI-2, Plasminogen Activator Inhibitor-2; PGE_2_, Prostaglandin E_2_; IFN-γ, Interferon Gamma*.

### Dental Caries

#### Background

A definition often used to describe dental caries (i.e., tooth decay or cavities) is: “the localized destruction of susceptible dental hard tissues (enamel, dentine, root cementum) by acidic by-products from bacterial fermentation of dietary carbohydrates” (178). It can be subdivided into coronal caries (affecting the crown portion of the tooth) and root caries (affecting the root of the tooth). People are susceptible to caries throughout their entire life. The increasingly older western population shows an increase in retention of their natural dentition and therefore, these individuals remain susceptible for caries development, also at old age. Hence, an increase in prevalence of dental caries is observed in this age category (179). Despite this, a clear drop in the total prevalence of caries has been observed in developed countries over the last few decades (178, 180, 181). Nevertheless, the earlier mentioned “G*lobal burden of disease 2010 study”* estimates a global prevalence of 35% for dental caries, ranking this oral disease first on the list of the most common diseases of mankind (79, 80).

As can be deduced from the earlier mentioned definition, on the one hand, dental caries is the result of a complex interaction between acid producing bacteria and fermentable carbohydrates (such as: sucrose, fructose, and glucose). On the other hand, the ecology of the mouth, in this case determined to a great extent by the pH of saliva and competing non-cariogenic bacteria, is also important. In the healthy situation, there is a balance between the bacterial biofilm and the tooth minerals. If this balance is disturbed (often referred to as the ecological shift), bacteria in the dental biofilm (including *Streptococcus mutans*, other closely related *Streptococci* and some *Lactobacilli*) produce a sticky polysaccharide matrix and acids, causing demineralization of the hard dental tissues. Ultimately, this leads to cavitation. As human teeth are composed of non-shedding tissue, the aciduric bacteria can prolong this activity as long as they remain on the surface and have sufficient nutrition, in particular sucrose. Thus, poor oral hygiene and very regular intake of sucrose-rich or other fermentable carbohydrates-containing food, snacks, and soft-drinks—that also may be acidic—are cariogenic lifestyle habits. The process of demineralization can be stopped or even reversed, as there is a balance between demineralization and remineralization. This balance is influenced by the supply of calcium, phosphate, and fluoride, the composition and quantity of saliva (which serves as a buffer), diet and of course the presence of cariogenic bacteria in the dental plaque. In this way, physical, biological, environmental, and behavioral risk factors are involved in caries development (178).

#### Relationship With Diabetes Mellitus: Epidemiology

Several literature reviews that discuss the association between diabetes and dental caries, point to the lack of solid evidence for a clear correlation (6, 93, 182–184). The majority of studies have a cross-sectional design, investigating patients with T1DM. In many studies, these patients showed an increased prevalence of dental caries, compared to patients without DM (185–194). However, a considerable number of studies could not replicate these findings (195–201).

There is limited research available on the epidemiology of caries and T2DM. Some papers found an increased prevalence of dental caries (202–206), which was supported by investigations using diabetic animal models (207–209). On the contrary, several studies did not find a significant association between T2DM and caries (197, 210–212).

Longitudinal research with children and adults into the effect of DM on the development of dental caries is very rare. A prospective cohort study reported that poor glycemic control was associated with increased caries incidence within T1DM children (192). No longitudinal studies are available on patients with T2DM.

In general, the cross-sectional design of most studies, varying definitions of dental caries that limit generalizability, and possible differences between the mostly young T1DM and the generally older T2DM population, make it difficult to establish a clear epidemiologic association between DM and dental caries. It is worth noting that some studies even report a decreased prevalence of dental caries in patients with DM (213, 214). It can be speculated that this remarkable finding could be ascribed to proper diabetes care management. As we know, lifestyle adjustment and losing weight are important in diabetes care, especially for T2DM. Nutritional management (i.e., restrictions of carbohydrate intake) is a crucial part of the strategy to lose weight. For T1DM, restrictions in carbohydrate intake could be a strategy to achieve reduction in insulin doses. The relatively low, and/or more infrequent, intake of fermentable carbohydrates might reduce the risk for developing dental caries (93).

#### Relationship With Diabetes Mellitus: Pathogenesis

In contrast to periodontal disease, less is known about biologic explanations for the possible association between DM and dental caries. Nevertheless, we will review the same five pathologic mechanisms we discussed before. However, it is important to note several complicating factors. First, adult and elderly individuals with DM often suffer from periodontitis as discussed before. This is associated with gingival recession (i.e., receding gums), which exposes dental root surfaces that then become susceptible to root caries (183). As we will discuss in section Dry Mouth below, patients with DM also often suffer from decreased salivary flow rates and altered saliva composition. Since saliva acts as a buffer against the acidic by-products from the bacterial fermentation of carbohydrates, a change in mere quantity (i.e., hyposalivation) or composition of saliva could therefore influence that protective function against dental caries (203). For example, low salivary calcium and phosphate levels are associated with higher caries experience (215). Two studies indeed found lower calcium and phosphate levels in patients with DM, which was associated with higher caries prevalence (203, 204).

##### Hyperglycemia

Only a limited number of studies investigated the direct effect of hyperglycemia on dental caries. One animal study with diabetic rats showed that prevention of hyperglycemia by insulin treatment prevented the progression of dental caries (216). Studies with human subjects with either T1DM or T2DM found that an increased number of decayed teeth was associated with higher HbA_1c_ levels (217, 218). A large Asian adult cohort investigated the relationship between dental caries and metabolic syndrome (219). Metabolic syndrome is defined by having three of the following five components: hyperglycemia, elevated triglycerides, lowered HDL levels, hypertension, and central obesity. Multivariate analysis on these individual components revealed a significant association between dental caries and hyperglycemia (219). Another study also found a significant association between dental caries and metabolic syndrome, but no relationships with the individual components were observed (220). Reports about the downstream pathways of hyperglycemia (polyol, AGEs, PKC, hexosamine) and upstream effects (ROS production) in relation to caries are lacking.

Interestingly, hyperglycemia results in increased levels of glucose in saliva of patients with DM (221). This could be important, as glucose might serve as a source of nutrition for the cariogenic bacteria in the dental biofilm. Indeed, some studies found an association between these increased salivary glucose levels and dental caries in patients with DM (192, 222), however, others did not (193, 196). Therefore, at this point, there is inconclusive evidence that salivary hyperglycemia is associated with increased dental caries.

##### Insulin resistance

One study compared the decayed, missing, and filled teeth (DMFT) index—a marker for experienced and current caries—between obese, insulin-resistant (OB-IR) patients and healthy controls. The DMFT index, as well as one of its components (decayed teeth), were increased in patients with insulin resistance (223).

##### Dyslipidemia

Research into the involvement of dyslipidemia in dental caries is limited to those studies we mentioned above, which investigated dyslipidemia as a component of metabolic syndrome. None of those found a relationship, as only hyperglycemia was significantly associated with decayed teeth (219, 220).

##### Hypertension

A recent study with indigenous Brazilian adolescents revealed that hypertension was significantly associated with dental caries (OR = 1.95, 95% CI: 1.03–3.66) (224). Another study found a significant association between and dental caries—measured as DMFT and DMFS (Decayed, Missing due to caries, Filled teeth/Surface)—and self-reported hypertension (225).

##### Immune dysfunction: impaired immune response and proinflammatory state

There is no evidence that inflammation plays a role in dental caries, as is the case for periodontitis or the chronic systemic complications of DM. However, the impaired immune defense against opportunistic pathogens might influence the number of cariogenic bacteria in saliva and dental biofilm. Increased counts of streptococci and lactobacilli were observed in the supragingival plaque (the biofilm on teeth along and above the gum margins) of patients with DM, which was associated with increased caries incidence (213, 226). Another study showed that, especially in persons with poorly controlled DM, the presence of caries was associated with high levels of lactobacilli and *Streptococcus mutans*, compared to patients with well-controlled DM (199). One study proposed that elevated levels of salivary glucose—presumably caused by obesity or DM—favors a relative abundance of aciduric bacteria that increases the risk for developing dental caries (227). Others did not find any differences in cariogenic bacteria in saliva between patients with and without DM (210).

#### Concluding Comments

Summarizing the findings of the studied literature in Table 2, it becomes clear that several studies observe an increased prevalence of dental caries in patients with DM. Although it is conceivable that caries and DM are associated, the lack of longitudinal research prevents us from making causal assumptions. Moreover, it is not clear whether the increased prevalence is a direct result of DM, or that other factors contribute to the association. For example, certain lifestyle aspects, such as an unhealthy diet with high carbohydrates intake, increases the risk for DM as well as for dental caries.

**Table 2:** Overview of studies investigating the association between diabetes mellitus and dental caries.

**Study**	**Study population**	**Study design**	**Relationship?**	**Authors' conclusions**
**EPIDEMIOLOGY**				
Alves et al. (195)	Group 1: Patients with T1DM (*n =* 51; 56% male, 44% female; mean age = 11.3 ± 3.4 years). Group 2: Control subjects without DM (*n =* 51; 37% male, 63% female; mean age = 11.9 ± 3.4 years).	Cross-sectional study	*No*	No significant difference was found in DMFT and def-t between patients with DM and controls.
Arheiam and Omar (185)	Group 1: T1DM children (*n =* 70; 45 males, 25 females; mean age = 11.66 ± 1.44 years). Group 2: Control subjects without DM (*n =* 70; 45 males, 25 females; mean age = 11.63 ± 1.54 years).	Cross-sectional study	*Yes*	Children with DM showed higher means for the amount of decayed teeth and missing teeth, compared to healthy controls.
Saes Busato et al. (194)	Group 1: Patients with T1DM (*n =* 51; 24 males, 27 females; mean age = 17 years, range 14–19 years). Group 2: Age- and gender matched control subjects without DM (*n =* 51).	Cross-sectional study	*Yes*	A significantly higher DMF index was observed in patients with DM, compared to controls. No influence of metabolic control was found, possibly caused by the small sample size.
Cherry-Peppers and Ship (186)	Group 1: Patients with T2DM (*n =* 11; mean age = 67.9 ± 11.1 years). Group 2: Patients with impaired glucose tolerance (*n =* 32; mean age = 60.7 ± 19.1 years). Group 3: Control subjects without DM (*n =* 43; mean age = 60.2 ± 16.8 years).	Cross-sectional study	*Yes*	More coronal caries were present in patients with DM compared to the other groups.
Collin et al. (210)	Group 1: Patients with T2DM (*n =* 25; 3 males, 22 females; mean age = 67 years, range 58–76 years). Group 2: Control subjects without DM (*n =* 40; 21 males, 19 females; mean age = 66 years, range 59–77 years).	Cross-sectional study	*No*	No difference in prevalence of root and coronal caries was observed in patients, compared to controls.
Edblad et al. (187)	Group 1: Young adults with T1DM (*n =* 41; 15 males, 26 females; mean age = 21 ± 1.6 years). Group 2: Age- and gender matched healthy controls (*n =* 41).	Cross-sectional study	*Yes*	Patients with DM showed more initial buccal caries, compared to the healthy controls.
Gómez-Díaz et al. (188)	Patients with T1DM (*n =* 69; 36 males, 33 females; range 6–17 years old), subdivided in: Group 1: Well controlled (HbA_1c_ ≤ 7.0%). Group 2: Moderately controlled (HbA_1c_ 7.1–8.5). Group 3: Poorly controlled (HbA_1c_ >8.5%).	Cross-sectional study	*Yes*	More buccodental conditions were found in patients with poorly controlled DM (HbA_1c_ >8.5%).
Hintao et al. (202)	Group 1: Patients with T2DM (*n =* 105; 50.5% female; mean age = 54.3 ± 8.7 years). Group 2: Control subjects without DM (*n =* 103; 50.5% female; mean age = 53.3 ± 7.6 years).	Cross-sectional study	*Yes*	Patients had a higher prevalence of root surface caries and a higher number of decayed or filled root surfaces compared with subjects without DM.
Jawed et al. (204)	Group 1: Patients with T2DM (*n =* 400; mean age = 41.0 ± 9.7 years). Group 2: Age- & gender matched controls without DM (*n =* 300; mean age = 41.6 ± 11.3 years).	Cross-sectional study	*Yes*	A higher DMFT score was observed in patients, compared to controls. The role of glycemic control, salivary flow and salivary calcium level were pointed out.
Jawed et al. (203)	Group 1: Patients with T2DM (*n =* 398; 198 males, 200 females; mean age = 40.2 ± 9.0 years). Group 2: Healthy controls without DM (*n =* 395; 194 males, 201 females; mean age = 39.9 ± 10.6 years).	Cross-sectional study	*Yes*	A higher DMFT score was seen in patients, compared to controls. Also, a role of glycemic control was observed.
Johnston and Vieira (225)	1,281 subjects, aged 6–94 years (mean age = 47.71 years). No data about the number of subjects with DM.	Cross-sectional study	*Yes*	Cross-sectional analysis of the subjects regarding caries experience and systemic diseases revealed a significant association between dental caries and DM.
Jones et al. (205)	Group 1: Adults with non-specified DM (*n =* 642; 78% dentate). Group 2: “General population”, represented by the Adult Dental Health Survey (ADHS) (*n =* 916; 77% dentate).	Cross-sectional study	*Yes*	Patients suffered from a higher rate of caries than controls.
Kodama et al. (208)	Group 1: Type 1 diabetic rats (type: WBN/KobSlc). Group 2: Age- and gender matched non-diabetic rats (type: F344).	Longitudinal animal study	*Yes*	The diabetic conditions in the rats lead to elevated and more advanced caries development, compared to the non-diabetic rats.
Lin et al. (212)	Group 1: Patients with T2DM (*n =* 24; 10 males, 14 females; mean age = 71 years, range 54–85 years). Group 2: Controls without DM (*n =* 18; 10 males, 8 females; mean age = 73 years, range 57–86 years).	Cross-sectional study	*No*	After correction for the available teeth surfaces, the prevalence of coronal and root-surface caries was not increased in patients with DM.
Marín et al. (197)	Group 1: Patients with T1DM (*n =* 35; HbA_1c_ of 6.5–7%). Group 2: Patients with T1DM (*n =* 35; HbA_1c_ >7%). Group 3: Controls without T1DM (*n =* 35). Group 4: Patients with T2DM (*n =* 35). Group 5: Controls without T2DM (*n =* 35).	Cross-sectional study	*No*	No differences between patients with DM and controls were found with regard to dental caries.
Miko et al. (189)	Group 1: Patients with T1DM (*n =* 259; aged 14–19 years old). Group 2: Age- and gender matched control subjects without DM (*n =* 259).	Cross-sectional study	*Yes*	A higher DMFT and an increased number of filled teeth were found in patients with T1DM, compared to the control group. The number of decayed teeth was lower in patients with DM, which the authors ascribe to early dentist consultation and treatment of caries in that group.
Miralles et al. (190)	Group 1: Patients with T1DM, (*n =* 90; aged 18–50 years old). Group 2: Matched controls without DM (*n =* 90).	Cross-sectional study	*Yes*	DM patients showed a higher prevalence (authors incorrectly report incidence) of caries (measured as CAO index). Poor metabolic control (HbA1c >7.5%), disease duration and the presence of other complications were not associated with caries.
Moore et al. (196)	Group 1: Patients with T1DM (*n =* 390; 199 males, 191 females; mean age = 32.6 ± 0.4 years). Group 2: Controls (*n =* 202; 76 males, 126 females; mean age = 33.0 ± 0.5 years).	Cross-sectional study	*No*	Patients with DM did not show higher DFS compared to controls. A slightly increased RDFS was observed, which was associated with periodontal disease at older age.
Rai et al. (191)	Group 1: T1DM children (*n =* 100, aged 6–12 years). Group 2: Matched children without DM (*n =* 100).	Cross-sectional study	*Yes*	A higher caries prevalence (authors incorrectly report incidence) was seen in patients with DM, compared to healthy controls.
Sandberg et al. (206)	Group 1: Patients with T2DM (*n =* 102; 64 males, 38 females; mean age = 64.8 ± 8.4 years). Group 2: Control subjects without DM (*n =* 102; 64 males, 38 females; mean age = 64.9 ± 8.5 years).	Cross-sectional study	*Yes*	Patients with DM displayed an increase in initial caries lesions compared to the controls without DM, as well as a larger need for caries prevention.
Sano et al. (209)	Group 1: Type 2 diabetic mice (*db/db*, 9 males, 11 females). Group 2: Non-diabetic mice (*db/+*, 9 males, 9 females).	Cross-sectional animal study	*Yes*	The diabetic mice showed a higher prevalence of dental caries, compared to the control mice.
Swanljung et al. (198)	Group 1: Patients with T1DM (*n =* 85; mean age = 15.1 ± 1.5 years). Group 2: Age- & gender matched controls (*n =* 85; mean age = 15.1 ± 1.6 years).	Cross-sectional study	*No*	The patients with DM did not show a significantly higher DMF, DMFS or initial caries lesions compared to the controls. Important note: all patients with DM were well regulated.
Tagelsir et al. (200)	Group 1: Patients with T1DM (*n =* 52; 29 males, 23 females; aged 3–16 years). Group 2: Matched healthy controls (*n =* 50; 28 males and 22 females; aged 2–16 years).	Cross-sectional study	*No*	There was no significant difference in caries experience between patients and controls.
Tavares et al. (201)	Group 1: Patients with T1DM (*n =* 88; 61.1% male, 38.9% female; mean age = 55.7 ± 7.1). Group 2: Controls without DM (*n =* 185; 47.6% male, 52.4% female; mean age = 56.3 ± 5.2).	Cross-sectional	*No*	No significant differences in numbers of buccal surface sites with gingival recession or in numbers of root caries were found between the patients with DM and the control subjects.
Yeh et al. (207)	Group 1: Type 1 diabetic mice (*Ins2* Akita mice, i.e., Akita–/–). Group 2: Age- and gender matched wild-type control mice (Akita +/+).	Longitudinal animal study	*Yes*	A strong link between glycemic control and salivary dysfunction was found. The combination of hyperglycemia and hyposalivation had a negative influence on enamel mineralization, increasing the risk for tooth decay.
**PATHOGENESIS**				
**Hyperglycemia**				
Bakhshandeh et al. (218)	Dentate patients with DM (both T1DM and T2DM; *n =* 299; mean age = 49 ± 7.6 years).	Cross-sectional study	*Yes*	An association between metabolic control (HbA_1c_ >8.5%). and duration of DM (≥ 7 years) and mean DMFT was found in men, not in women.
Cao et al. (219)	Group 1: Patients with MetS (*n =* 3,571; 70.9% male, 29.1% female; mean age = 53.7 ± 5.9 years). Group 2: Patients without MetS (*n =* 10,427; 55.4% male, 44.6% female; mean age = 52.4 ± 5.8 years).	Cross-sectional study	*Yes*	After adjustment for multiple confounders, stratified regression analysis showed an association between dental caries and hyperglycemia as part of MetS (OR = 1.14, 95% CI: 0.98–1.34).
Siudikiene et al. (213)	Group 1: Patients with T1DM (*n =* 68, aged 10–15 years old). Group 1a: Patients with well-to-moderately controlled T1DM (*n* = 39; HbA_1c_ < 9%). Group 1b: Patients with poorly controlled T1DM (*n* = 29; HbA_1c_ ≥9.0%). Group 2: Age- & gender matched controls (*n =* 68).	Cross-sectional study	*Unclear*	In the total population, patients with DM even showed fewer caries compared to the controls. However, amongst patients with DM, well-to-moderately regulated patients had fewer caries, compared to poorly regulated patients.
Syrjälä et al. (199)	Patients with T1DM (*n =* 149; 87 males, 62 females; mean age = 34.1 ± 12.4 years).	Cross-sectional study	*No*	HbA_1c_ levels were not directly associated with caries prevalence. However, in poorly regulated patients (HbA_1c_ >8.5%), a positive association of mutans streptococci and lactobacilli with dental caries was found.
Timonen et al. (200)	Group 1: Patients with MetS (*n =* 337; 50.7% male, 49.3% female; mean age = 48.5 ± 0.5 years). Group 2: Subjects without MetS (*n =* 1,713; 37% male, 63% female; mean age = 45.5 ± 0.2 years).	Cross-sectional study	*No*	Despite a significant association between MetS as a whole and dental caries, no independent association was found between hyperglycemia (as a component of MetS) and caries after multiple adjustments.
Twetman et al. (192)	Children with T1DM (*n =* 64; 32 males, 32 females; mean age = 11.2 years, range 8–15 years).	Prospective cohort study	*Yes*	Children with poor glycemic control (HbA_1c_ >8.0%) showed a higher 3-year incidence of caries, compared to children who were well controlled (HbA_1c_ ≤ 8.0%). Other risk factors included: poor oral hygiene, previous caries experience and high levels of salivary lactobacilli.
Yonekura et al. (217)	Group 1: Patients with well-controlled T2DM (*n =* 24; 15 males, 9 females; mean age = 64.5 years). Group 2: Patients with poorly controlled T2DM (*n =* 84; 48 males, 36 females; mean age = 59.0 years).	Cross-sectional study	*Yes*	The prevalence of decayed teeth (DT) was higher in patients with poorly controlled T2DM. Logistic regression analysis showed significant association between HbA_1c_ levels and the absolute number of DT.
**Insulin resistance**				
Loyola-Rodriguez et al. (223)	Group 1: Patients with obesity and insulin resistance (*n =* 50; 23 males, 27 females; mean age = 13.0 ± 1.2 years). Group 2: Healthy controls (*n =* 50; 21 males, 29 females; mean age = 13.1 ± 1.1 years).	Cross-sectional study	*Yes*	Obese patients with insulin resistance showed a higher DMFT index and number of decayed teeth, compared to the control group. A multivariate regression analysis revealed odds ratios for insulin resistance of 3.1 for DMFT index and 3.3 for decayed teeth.
Timonen et al. (200)	Group 1: Patients with MetS (*n =* 337; 50.7% male, 49.3% female; mean age = 48.5 ± 0.5 years). Group 2: Subjects without MetS (*n =* 1713; 37% male, 63% female; mean age = 45.5 ± 0.2 years).	Cross-sectional study	*No*	Despite a significant association between MetS as a whole and dental caries, no independent association was found between insulin resistance (as a component of MetS) and caries after multiple adjustments.
**Dyslipidemia**				
Cao et al. (219)	Group 1: Patients with MetS (*n =* 3,571; 70.9% male, 29.1% female; mean age = 53.7 ± 5.9 years). Group 2: Patients without MetS (*n =* 10,427; 55.4% male, 44.6% female; mean age = 52.4 ± 5.8 years).	Cross-sectional study	*No*	After adjustment for multiple confounders, stratified regression analysis showed no association between dental caries and dyslipidemia (OR = 1.01 for hypertriglyceridemia [95% CI: 0.85–1.19] and 0.84 for low HDL-c [95% CI: 0.70–1.00]).
Timonen et al. (200)	Group 1: Patients with MetS (*n =* 337; 50.7% male, 49.3% female; mean age = 48.5 ± 0.5 years). Group 2: Subjects without MetS (*n =* 1713; 37% male, 63% female; mean age = 45.5 ± 0.2 years).	Cross-sectional study	*No*	Despite a significant association between MetS as a whole and dental caries, no independent association was found between dyslipidemia (as a component of MetS) and caries after multiple adjustments.
**Hypertension**				
Cao et al. (219)	Group 1: Patients with MetS (*n =* 3,571; 70.9% male, 29.1% female; mean age = 53.7 ± 5.9 years). Group 2: Patients without MetS (*n =* 10,427; 55.4% male, 44.6% female; mean age = 52.4 ± 5.8 years).	Cross-sectional study	*No*	After adjustment for multiple confounders, stratified regression analysis showed no association between dental caries and hypertension (OR = 0.96; 95% CI: 0.86–1.13).
Johnston and Vieira (225)	1,281 subjects, aged 6–94 years (mean age = 47.71 years). No data about the number of subjects with DM.	Cross-sectional study	*Yes*	Both primary (DMFT) and secondary (DMFS) caries experience were independently associated with hypertension.
Ribeiro et al. (224)	Adult subjects (*n =* 225, 101 males, 124 females; 60.9% aged 19–34, 39.1% aged ≥35).	Cross-sectional study	*Yes*	Patients with hypertension (*n =* 54) had a higher prevalence of dental caries (55.6%), compared to subjects without hypertension (*n =* 171, 38.6%). Logistic regression analysis revealed an independent association between hypertension and dental caries (OR = 1.95; 95% CI: 1.03–3.66)
Timonen et al. (200)	Group 1: Patients with MetS (*n =* 337; 50.7% male, 49.3% female; mean age = 48.5 ± 0.5 years). Group 2: Subjects without MetS (*n =* 1713; 37% male, 63% female; mean age = 45.5 ± 0.2 years).	Cross-sectional study	*No*	Despite a significant association between MetS as a whole and dental caries, no independent association was found between hypertension (as a component of MetS) and caries after multiple adjustments.
**Immune dysfunction**				
Collin et al. (210)	Group 1: Patients with T2DM (*n =* 25; 3 males, 22 females; mean age = 67, range 58–76). Group 2: Control subjects without DM (*n =* 40; 21 males, 19 females; mean age = 66, range 59–77).	Cross-sectional study	*No*	There was no difference in cariogenic microorganisms and yeasts between patients with or without DM. It should be noted that they also did not find a higher prevalence of dental caries.
Goodson et al. (227)	Adolescents (*n =* 8,173; 38.9% male, 61.1% female; mean age = 10.0 ± 0.7 years).	Cross-sectional study	*Yes*	Increasing salivary glucose levels—presumably caused by obesity and/or DM—reduced the total bacterial load in saliva. The authors hypothesize that glucose metabolism by acidogenic bacteria lowers the salivary pH, which disturbs the oral microbiome and favors cariogenic bacteria species.
Kampoo et al. (226)	Group 1: Patients with T2DM (*n =* 20; 5 males, 15 females; mean age = 56.4 years). Group 2: Control subjects without DM (*n =* 11; 4 males, 7 females; mean age = 37.1 years).	Cross-sectional	*Yes*	Patients with DM showed higher numbers of total streptococci and lactobacilli in supragingival plaque, compared to the control subjects. Lactobacillus numbers were also increased in saliva and supragingival plaque of patients with DM and active caries, compared to patients with DM, without active caries.
Siudikiene et al. (213)	Group 1: Patients with T1DM (*n =* 68, aged 10-15 years old). Group 2: Age- & gender matched controls (*n =* 68).	Cross-sectional study	*Yes*	Patients with DM showed more frequent presence of yeasts, and poorly controlled patients also had higher numbers of mutans streptococci.
Syrjälä et al. (199)	Patients with T1DM (*n =* 149; 87 males, 62 females; mean age = 34.1 ± 12.4)	Cross-sectional study	*Yes*	In poorly regulated patients (HbA_1c_ ≥8.5%), a positive association of mutans streptococci and lactobacilli with dental caries was found.

*T1DM, Diabetes Mellitus Type 1; T2DM, Diabetes Mellitus Type 2; DMF(T/S), Decayed, Missing, Filled (Teeth/Surfaces); Def-t, Decayed, extracted, filled tooth index; CAO, Caried, Absent and Obturated teeth index; (R)DFS, (Root) Decayed and Filled Surfaces; MetS, Metabolic Syndrome; DT, Decayed Teeth*.

### Dry Mouth

#### Background

The term “dry mouth” can be interpreted in several ways. Usually, two distinctive phenomena are referred to in literature when speaking of dry mouth. The first is the *objective* measurement of reduced salivary flow rate, called hyposalivation or salivary gland hypofunction. This is usually defined as “an unstimulated whole saliva flow rate of < 0.1 mL/min, collected for 5 to 15 min, or chewing-stimulated whole saliva flow rate of < 0.7 mL/min, collected for 5 min” (228). The second condition often referred to as “dry mouth” is xerostomia, which addresses the *subjective* experience of a dry mouth by the patient (229). Even though these conditions are closely related, patients with hyposalivation do not necessarily suffer from xerostomia. And *vice versa*, although many patients with xerostomia do actually have underlying hyposalivation, this is not always the case. This suggests that other mechanisms could partly explain the dry mouth sensation in some patients, such as a change in saliva composition, a sensory dysfunction or a cognitive problem (230–232).

When defining hyposalivation as an unstimulated whole saliva flow rate of < 0.1 mL/min, research showed that approximately 20% of individuals across all ages were affected, while the prevalence was ~3% when using the stimulated whole saliva flow rate definition (< 0.7 mL/min) (233). The prevalence of xerostomia is subject of discussion, as estimations in literature range from 1 to 65% (234). This wide range is mainly the result of high variation in defining xerostomia and methods of diagnosis, and implies that there is need for large population-based research. However, it is clear that the prevalence of both conditions increases with age (234). Furthermore, medication use is one of the major causes of both xerostomia and hyposalivation (235), as well as Sjögren syndrome and radiation therapy of the head and neck region (229). Xerostomia is also associated with several autoimmune diseases, graft-vs.-host disease after undergoing bone marrow transplantation, HIV infection, and renal failure (236).

#### Relationship With Diabetes Mellitus: Epidemiology

The experience of a dry mouth is one of the most frequently mentioned oral complaints by patients with DM. In a way, this is not surprising, considering the fact that the population with T2DM generally consists of relatively older individuals, and we now know that the prevalence of xerostomia and hyposalivation increases with age. Also, many patients with DM suffer from one or more complications and/or comorbidities, for which they possibly receive medication that increases the risk for oral dryness (e.g., anti-cholinergic and anti-hypertensive medication).

Varying definitions and methods of diagnosing hyposalivation and xerostomia make it difficult to establish a direct association with DM. Nevertheless, many cross-sectional studies reported a diminished salivary flow rate in patients with DM, compared to control subjects without DM. This finding applies for patients with T1DM (237–243), as well as for individuals with T2DM (204, 206, 239, 241, 244–252). However, it should be noted that in several of these studies, the measured decrease in salivary flow rate did not necessarily fulfill the earlier mentioned criteria to be classified as hyposalivation. This could be one of the reasons why some studies did not find an increased prevalence of xerostomia in patients with T1DM (237, 253) or T2DM (243, 244, 253). This is supported by the suggestion that in general, a salivary flow rate above the threshold of 0.1–0.3 mL/min is sufficient to prevent xerostomia in most individuals (254). Still, there is sufficient literature that reports an increased prevalence of xerostomia in patients with T1DM (240, 241, 243, 255–257) and T2DM (206, 247, 248, 257, 258).

#### Relationship With Diabetes Mellitus: Pathogenesis

Although many cross-sectional studies report an increased prevalence of xerostomia and a decreased salivary flow rate in patients with DM, the mechanisms underlying these observations remain unclear. Secretion of saliva by the parotid gland—the largest salivary glands—is regulated by the autonomic nervous system, which led to the hypothesis that diabetic neuropathy might somehow be involved in the development of dry mouth experience. However, studies investigating this theory present contradictory results. Decreased (240, 259), comparable (241, 260), or even increased (261) salivary flow rates are observed in patients with DM, having peripheral or autonomic neuropathy, compared to subjects without DM and/or patients with DM without neuropathy. It should be noted that tricyclic anti-depressants, sometimes prescribed to relieve neuropathic pain, are associated with the development of dry mouth and therefore is another xerogenic drug (235).

There are a few studies that actually observed structural changes in the salivary glands of patients with DM, in particular in the parotid glands. Vacuolization of acini (the saliva secretory cells) in the parotid gland was observed, which indicated an early form of degeneration (262). Another study also showed atrophy of the acini, lipid droplets in epithelial cells of the acini and ducts, and adipose infiltration in the stromae (263). On the contrary, a study into the submandibular gland of patients with T2DM observed enlargement of the acini, independent of impaired glandular function or metabolic control. This might indicate a compensatory mechanism to counteract hyposalivation (264). Asymptomatic enlargement of the parotid gland is also frequently observed in patients with DM, which could indicate similar compensatory mechanisms against hyposalivation or xerostomia (265). The pathological mechanisms of how DM contributes to these structural changes are still matter of discussion, and solid research is scarce. Below, we summarize possible mechanisms of action how DM might induce xerostomia or hyposalivation, again via the established pathological mechanisms of diabetic complications.

##### Hyperglycemia

In general, patients with poor glycemic control present significantly lower salivary flow rate and higher prevalence of xerostomia, compared to subjects with well-controlled DM (240, 241, 244, 245). This suggests that, again, hyperglycemia is a driving force in the pathogenic association between DM and dry mouth. However, the downstream pathways of hyperglycemia (polyol, AGEs, PKC, and hexosamine) are completely unexplored in human study subjects, as well as the common upstream effect of increased oxidative stress. One animal study suggests that the expression of AGEs and RAGE in the lacrimal glands (which secrete the aqueous layer of the tear film) might cause eye dryness (266). Theoretically, that could also account for the salivary glands, causing oral dryness. Indeed, submandibular salivary glands of diabetic rats also showed increased expression of RAGE, which resulted in an inflammatory upregulation through NF-κB activation (267). However, this hypothesis has not been studied in humans so far and demands further research.

Hyperglycemia in patients with DM can cause polyuria and osmotic diuresis, possibly leading to dehydration, which is associated with hyposalivation (268, 269). Also, a fairly novel class of antidiabetic drugs (SGLT-2 inhibitor) prevents reabsorption of glucose in the kidneys, and thereby increases urinary glucose excretion (i.e., glycosuria). This could lead to dehydration and increased thirst, both associated with oral dryness (270). Other xerogenic medications that patients with DM often need (e.g., anti-hypertensives) might also partly explain the association between DM, hyperglycemia and reduced salivary flow or xerostomia (241).

##### Insulin resistance

Research into the role of insulin resistance in the pathogenesis of hyposalivation and xerostomia is limited to a few animal studies. Increased ROS production, an upregulated inflammatory response (271) and an altered lipid profile (272) were observed in the submandibular and parotid salivary gland of insulin resistant rats. The pro-inflammatory state observed in salivary glands did not alter their structural morphology (273).

##### Dyslipidemia

As we mentioned earlier, some studies observed an accumulation of lipid droplets in epithelial cells of the salivary glands (263, 264). This might be the consequence of the excess flux of free fatty acids (FFAs), resulting from insulin resistance (see section Insulin Resistance). In several tissue types, accumulation of lipids induces lipotoxicity, which can lead to cell apoptosis (274). It is hypothesized that these processes could also affect the salivary glands and thereby cause hyposalivation, but this theory has not been investigated yet (264). As a matter of fact, to the best of our knowledge, the independent role of dyslipidemia in the association between xerostomia/hyposalivation and DM has not been investigated in humans. One animal study showed that saturated fatty acids (SFAs) induced an inflammatory response, reflected by increased production of IL-6 through NF-κB activation in the salivary glands epithelial cells (275).

##### Hypertension

One study showed that patients with hypertension and without DM had reduced submandibular and sublingual salivary flow rates, compared to healthy controls. The salivary flow rates of the hypertensive patients were comparable with those of patients with DM, while stimulated parotid and unstimulated whole salivary flow did not differ across the groups. However, it was not clear whether these differences were a consequence of the hypertension itself, possible anti-hypertensive medication use, or both (276).

##### Immune dysfunction: impaired immune response and proinflammatory state

As described above, DM and its metabolic disturbances could be associated with dry mouth, sometimes mediated by inflammatory processes. However, this remains rather speculative, since most of those studies investigated diabetic animal models. Moreover, the inflammatory state observed in some studies did not necessarily affect salivary flow rate, xerostomia or salivary gland morphology.

Some studies suggest that Sjögren syndrome—an autoimmune disease affecting the salivary glands—could be the underlying cause of dry mouth in patients with DM (277, 278). However, there is no convincing evidence for this hypothesis.

#### Concluding Comments

Dry mouth is a very often heard complaint by patients with DM, and the majority of epidemiological studies indeed report an increased prevalence of xerostomia and a decreased salivary flow rate. Especially poor glycemic control negatively impacts both the prevalence and severity of dry mouth. Older age, dehydration and medication use also seem to be important determinants in this association. However, there is hardly any research into other pathogenic pathways that could explain the association between DM and dry mouth, and longitudinal studies are also lacking. Table 3 summarizes the literature on which this section was based.

**Table 3:** Overview of studies investigating the association between diabetes mellitus and dry mouth.

**Study**	**Study population**	**Study design**	**Relationship?**	**Authors' conclusions**
**EPIDEMIOLOGY**				
Ben-Aryeh et al. (237)	Group 1: Patients with T1DM (*n =* 35; 20 males, 15 females; mean age = 31.2 ± 7.4 years). Group 2: Control subjects without DM (*n =* 31; 17 males, 14 females; mean age = 29.0 ± 6.2 years).	Cross-sectional study	*Yes*	Patients with DM showed significantly lower salivary flow. Also, higher glucose, potassium and protein levels were seen in saliva of patients with DM, indicating affected salivary gland. No differences in xerostomia experience were observed.
Ben-Aryeh et al. (253)	Group 1: Patients with T1DM (*n =* 20; mean age = 38.9 ± 13.7 years). Group 2: Patients with T2DM (*n =* 19; mean age = 45.9 ± 11.2 years). Group 3: Healthy controls (*n =* 35; mean age = 40.4 ± 12.0 years).	Cross-sectional study	*No*	Patients with T1DM showed a significantly lower salivary flow compared to patients with T2DM, but not compared to controls. Potassium and IgA concentrations were different between the groups. No difference in the complaint of xerostomia was observed between the groups.
Busato et al. (255)	Group 1: Patients with T1DM (*n =* 51; 24 males, 27 females; mean age = 17 ± 1.4, range 14–19). Group 2: Age- and gender matched controls without DM (*n =* 51).	Cross-sectional study	*Yes*	DM appeared to be associated with a high prevalence of xerostomia, which in turn was predictive of a poor oral health related quality of life.
Carda et al. (258)	Group 1: Patients with T2DM (*n =* 17; 10 males, 7 females; mean age = 68, range 26–86 years). Group 2: Control subjects (*n =* 16; 8 males, 8 females; mean age = 48, range 26–86 years).	Cross-sectional study	*Yes*	Xerostomia was observed more often in patients with DM. Increased levels of urea and proteins were found in saliva of patients with DM, while albumin was decreased. Increased levels of salivary glucose were associated with poor metabolic control.
Ivanovski et al. (256)	Group 1: Patients with T1DM (*n =* 60). Group 2: Control subjects without DM (*n =* 60). Both males and females (proportion not given), aged 30–70 years.	Cross-sectional study	*Yes*	The authors conclude that patients with DM have significantly higher salivary levels of urea and glucose. Also, they claim diabetes causes xerostomia, and a significant correlation exists between the degree of xerostomia and the salivary glucose concentrations.
Jawed et al. (204)	Group 1: Patients with T2DM (*n =* 400; mean age = 40.9 ± 9.7 years). Group 2: Age- and gender matched controls (*n =* 300; mean age = 41.6 ± 11.3 years).	Cross-sectional study	*Yes*	Patients with DM had a lower salivary pH, flow rate, and calcium levels compared to controls. Fasting blood glucose, HbA_1c_, and DMFT score were significantly increased in patients with DM.
Kao et al. (246)	Group 1: Patients with T2DM and xerostomia (*n =* 20; 13 males, 7 females; mean age = 54.2 ± 14.5 years). Group 2: Patients with T2DM, without xerostomia (*n =* 20; 13 males, 7 females; mean age = 55.2 ± 13.4 years). Group 3: Healthy age- and gender matched controls (*n =* 36; 23 males, 13 females; aged 56.2 ± 13.3 years).	Cross-sectional study	*Yes*	Patients with DM and xerostomia (group 1) showed significantly lower saliva production and excretion, compared to the other two groups.
Karjalainen et al. (242)	Group 1: Newly diagnosed T1DM children (*n =* 14; 7 boys, 7 girls; mean age = 11 ± 2.4 years). Group 1: Patients with long-term T1DM (*n =* 50; 30 males, 20 females; mean age = 14.4 ± 1.7 years).	Cross-sectional	*Yes*	At initial T1DM diagnosis, hyperglycemia was reflected as increased levels of salivary glucose and a decreased salivary flow. Patients with long-term diabetes did not show such a clear relationship.
Khovidhunkit et al. (247)	Group 1: Patients with T2DM (*n =* 154; 37 males, 117 females; mean age = 63 ± 10 years). Group 2: Control subjects without DM (*n =* 50; 12 males, 38 females; mean age = 65 ± 10 years).	Cross-sectional study	*Yes*	A significantly higher prevalence of xerostomia and hyposalivation was observed in patients with DM, compared to the subjects without DM.
Lasisi and Fasanmade (252)	Group 1: Patients with T2DM (*n =* 20; 10 males, 10 female; mean age = 58.4 years). Group 2: Controls without DM (*n =* 20; 11 males, 9 female; mean age = 50.2 years).	Cross-sectional study	*Yes*	Patients with DM showed reduced salivary flow, and increased salivary concentrations of glucose and potassium. Total protein, Na^+^, Ca^2+^, Cl^−^, and ${\text{HCO}}_{3}^{-}$ concentrations did not differ.
Lin et al. (248)	Group 1: Patients with T2DM and xerostomia (*n =* 36; 23 males, 13 females; mean age = 56.3 ± 14.2 years). Group 2: Patients with T2DM, without xerostomia (*n =* 36; 23 males, 13 females; mean age = 56.0 ± 13.9 years). Group 3: Healthy controls (*n =* 36; 23 males and 13 females; mean age = 56.2 ± 13.3 years).	Cross-sectional study	*Yes*	Salivary scintigraphy revealed that salivary function (expressed as production and secretion) was significantly lower in patients with DM and xerostomia, compared to patients with DM, without xerostomia and healthy controls.
López et al. (238)	Group 1: Children with T1DM (*n =* 20; 9 males, 11 females; mean age = 9.4 ± 3.9 years). Group 2: Clinically healthy children (*n =* 21; 9 males, 12 females; mean age = 8.3 ± 1.8 years).	Cross-sectional study	*Yes*	Children with diabetes showed a lower salivary pH, a decreased flow, higher concentrations of sugars, glucose, urea, and total proteins and decreased levels of calcium, compared to the controls.
Malicka et al. (243)	Group 1: Patients with T1DM (*n =* 34; 16 males, 18 females; mean age = 37.5 years). Group 2: Patients with T2DM (*n =* 59; 31 males, 28 females; mean age = 65 years). Group 3: Healthy, age- and gender matched controls (*n =* 63).	Cross-sectional study	*Yes*	A significantly decreased salivary flow and increased prevalence of xerostomia were observed in patients with T1DM compared to controls. For patients with T2DM, these differences were not significant, but a trend was observed.
Mata et al. (239)	Group 1: Patients with T1DM (*n =* 30; age range 20–30 years) and patients with T2DM (*n =* 30; age range 40–55). Group 2: Two groups of age matched controls (2 times *n =* 30).	Cross-sectional study	*Yes*	Patients with DM showed a significant decrease in salivary flow and secretion capacity, while protein and Ca^2+^ levels were increased. Levels of Mg^2+^, Zn^2+^, and K^+^ were significantly lower compared to the control group.
Montaldo et al. (251)	Group 1: Patients with T2DM (*n =* 134; 56 males, 78 females; mean age = 47.9 ± 2.9 years). Group 2: Healthy controls (*n =* 111; 49 males, 62 females; mean age = 44.9 ± 5.8 years).	Cross-sectional study	*Yes*	Mean salivary flow appeared to be lower in the diabetes group, compared to the control group.
Quirino et al. (249)	Group 1: Patients with controlled T2DM (*n =* 35; 80% female, 20% male). Group 2: Patients with uncontrolled T2DM (*n =* 35, 68.7% female, 31.4% male).	Cross-sectional study	*Yes*	Hyposalivation was observed more often in patients with DM, compared to controls.
Sandberg et al. (260)	Group 1: Patients with T2DM (*n =* 102; 64% males, 36% females; mean age = 64.8 ± 8.4 years). Group 2: Age- & gender matched control subjects without DM (*n =* 102).	Cross-sectional study	*Yes*	Xerostomia was significantly more prevalent in patients with DM (53.5%) compared to the control group (28.4%).
Silveira Lessa et al. (257)	Group 1: Patients with T1DM and T2DM (combined *n =* 1,979). Group 2: Control subjects without DM (*n =* 1,225).	Systematic review and meta-analysis	*Yes*	The prevalence of xerostomia in patients with DM was higher (37.4% for T1DM and 46.1% for T2DM), compared to the control subjects without DM (24.2%). Furthermore, a significant association between DM and xerostomia was found when analyzing case-control studies (OR = 3.15, 95% CI: 2.11–4.70), indicating an increased risk for xerostomia when diabetes is present).
Vasconcelos et al. (250)	Group 1: Patients with T2DM (*n =* 40; 20 males, 20 females; mean age = 57.7 ± 8.9 years). Group 2: Control subjects without DM (*n =* 40; 20 males, 20 females; mean age = 50.2 ± 12.3 years).	Cross-sectional study	*Yes*	Both resting and stimulated salivary flow was lower in patients with DM, compared to the control group. However, no significant difference in the prevalence of xerostomia was found.
**PATHOGENESIS**				
**Hyperglycemia**				
Chávez et al. (244)	Group 1: Patients with better controlled T2DM (HbA_1c_ ≤ 9%) (*n =* 10; 5 males, 5 females; 6 ≤ 71 year old, 4 >71 years old). Group 2: Patients with poorly controlled T2DM (HbA_1c_ >9%) (*n =* 14; 5 males, 9 females; 6 ≤ 71 year old, 8 >71 years old). Group 3: Controls without DM (*n =* 15; 9 males, 6 females; 5 ≤ 71 year old, 10 >71 years old).	Prospective cohort study	*Yes*	Patients with poorly controlled diabetes showed lower stimulated parotid saliva flow rates, both at baseline and after 1 year follow-up. Also, patients with DM reported more complaints of thirst, though not of xerostomia. No changes over 1 year of follow-up were observed.
Chavez et al. (245)	Group 1: Patients with well controlled T2DM (HbA_1c_ ≤ 9%) (*n =* 11; 8 males, 3 females; 6 ≤ 71 year old, 5 >71 years old). Group 2: Patients with poorly controlled T2DM (HbA_1c_ >9%) (*n =* 18; 8 males, 10 females; 8 ≤ 71 year old, 10 >71 years old). Group 3: Patients without DM (*n =* 23; 9 males, 14 females; 9 ≤ 71 year old, 14 >71 years old).	Cross-sectional study	*Yes*	Patients with poorly regulated diabetes displayed a significant decrease in stimulated parotid salivary flow rates compared to well-controlled patients and subjects without DM.
Fukuoka et al. (267)	Group 1: Control rats (*n =* 11). Group 2: Diabetic rats (*n =* 9). Group 3: Diabetic rats, treated with low-power laser irradiation (*n =* 10). Diabetes was induced with Streptozotocin.	Longitudinal animal study	*Yes*	The diabetic rats showed an upregulated AGE/RAGE expression in the salivary glands, which resulted in an inflammatory response through activation of the NF-κB pathway. Therapy with the low-power laser irradiation suppressed this inflammatory state, and reduced apoptosis caused by diabetes.
Moore et al. (240)	Group 1: Patients with T1DM (*n =* 406; 204 males, 202 female; mean age = 33.0 ± 0.4 years). Group 2: Healthy controls (*n =* 268; 108 males, 160 female; mean age = 31.8 ± 0.5 years).	Cross-sectional study	*Yes*	Patients with DM complained more often of dry mouth and had decreased salivary flow rates compared to controls. Furthermore, decreased salivary flow rates were associated with increased fasting glucose levels.
Sreebny et al. (241)	Group 1: Patients with T1DM (*n =* 15) and T2DM (*n =* 25) Group 2: Age- and gender matched healthy control subjects without DM (*n =* 40).	Cross-sectional study	*Yes*	Patients with DM showed a decreased salivary flow compared to the control subjects. Also, an inverse relationship between metabolic control (HbA_1c_) and salivary flow was observed.
**Insulin resistance**				
Ittichaicharoen et al. (271)	Group 1: Normal diet fed Wistar rats (*n =* 8). Group 2: High-fat diet fed Wistar rats, vehicle treated (*n =* 8). Group 3: High-fat diet fed Wistar rats, vildagliptin treated (*n =* 8).	Longitudinal animal study	*Yes*	Rats fed with a high-fat diet developed obese-insulin resistance, with increased apoptosis, ROS production, inflammation and mitochondrial dysfunction in their salivary glands as a result. When treated with the anti-diabetic drug, these changes were reduced.
Matczuk et al. (272)	Group 1: Normal diet fed rats (*n =* 8) Group 2: High-fat diet fed rats (*n =* 8).	Longitudinal animal study	*Yes*	A high-fat diet, inducing insulin resistance and obesity, resulted in a changing lipid composition in rat salivary glands. Phospholipids and triacylglycerols were increased; FFAs and diacylglycerols were not.
Mozaffari et al. (273)	Group 1: Obese rats (*n =* 7). Group 2: Lean rats (*n =* 9). Rats used: male, obese (OBZ) and lean Zucker rats.	Cross-sectional animal study	*Unclear*	The obese rats develop insulin resistance, which resulted in an increased expression of ICAM-1 and an upregulation of the NF-κB pathway in the salivary glands. However, morphological changes were not observed.
**Dyslipidemia**				
Shikama et al. (275)	Human parotid and submandibular salivary gland epithelial cell lines, treated with various types of FFAs to mimic dyslipidemic conditions.	*Ex vivo* experiment	*Yes*	Treatment with saturated (not unsaturated) fatty acids (SFA) activates the NF-κB and MAPK pathway, resulting in increased IL-6 production in human parotid and submandibular salivary gland epithelial cell lines.
**Hypertension**				
Dodds et al. (276)	Group 1: Healthy controls (*n =* 240; 134 males, 116 females; mean age = 55.6 years). Group 2: Patients with hypertension (*n =* 227; 91 males, 136 females; mean age = 63.7 years). Group 3: Patients with DM (n = 233; 120 males, 113 females; mean age = 62.6 years).	Cross-sectional study	*Yes*	Patients with hypertension had lower unstimulated submandibular/sublingual (US) and stimulated submandibular/sublingual (SS) salivary flow rates, compared to healthy controls. US flow rates were also comparable with patients with DM.

*T1DM, Diabetes Mellitus Type 1; T2DM, Diabetes Mellitus Type 2; IgA, Immunoglobulin A; DMFT, Decayed, missing, filled teeth; (R)AGE, (Receptor for) Advanced Glycation End Products; NF-κB, Nuclear Factor Kappa B; FFA, Free Fatty Acids; ICAM-1, Intercellular Adhesion Molecule 1; MAPK, Mitogen-Activated Protein Kinase; IL-6, Interleukin 6*.

### Oral Mucosal Lesions

#### Background

The name oral mucosal lesion is used as an umbrella term for any abnormal change to the mucosal surface in the oral cavity. This concerns numerous different types of lesions, and it is beyond the scope of this review to discuss them all individually. Often, oral mucosal lesions are classified based on the pathology, morphology, or location of the lesion. Shulman et al. classify: “*candida*-related lesions, tobacco-related lesions, acute conditions, tongue conditions, red/white conditions, raised conditions, and other conditions.” Possible locations where mucosal lesions can be observed are: the hard palate, gingiva, lip, tongue, buccal mucosa, vestibule, labial mucosa, commissure, floor of the mouth, and the soft palate (279).

Despite the heterogeneity of oral mucosal lesions, several studies tried to estimate their prevalence. A large epidemiologic study from the U.S. (17,235 individuals >17 years old) concluded that 27.9% of the population had at least one lesion (279). In a German population, 33.8% of adults aged 35–44 years and 33.9% of adults aged 65–74 years were *without* any oral mucosal lesion (280). Somewhat similar figures were reported in a Slovenian population aged 25–75 years, where 38.4% had no oral mucosal lesions (281). A Chilean study showed a prevalence of 53% for one or more oral mucosal lesions, although in this study, only individuals older than 65 years were included (282). From the majority of studies, it can be concluded that tobacco use seems to be the most important risk factor for having any type of oral mucosal lesions, followed by (ill-fitting) removable dentures. The prevalence of oral mucosal lesions increases with age and is higher in males (279). Ethnicity might also play a role. For example, non-Hispanic whites in the US had increased odds of having a lesion, compared to non-Hispanic blacks and Mexican-Americans (279).

The large majority of studies investigating the association between DM and oral mucosal lesions focus on *Candida*-related lesions. For this reason, we will also focus especially on these forms of mucosal lesions, while we will only briefly discuss non-candidal oral mucosal lesions at the end of this section.

#### Relationship With Diabetes Mellitus: Epidemiology (*Candida*-Related Oral Lesions)

*Candida* is a genus of yeasts, with *Candida albicans* as the most common species, and it can be found on the skin and on all mucosal surfaces. It exists in healthy individuals as a commensal organism, but causes opportunistic infections in susceptible patients, such as patients with HIV/AIDS, persons using immunosuppressive medication and individuals with poorly controlled DM (283). Oral candidal infection (candidiasis) has several clinical manifestations, including *median rhomboid glossitis* (redness and loss of papillae on the dorsal side of the tongue), *angular cheilitis* (inflammation of the corners of the mouth) and *denture stomatitis* (inflammation and redness underneath a denture). Assessment of the association between DM and *Candida*-related lesions is hampered by the complexity of the lesions, variations in study populations and interaction with other local risk factors (284). Moreover, there are no longitudinal studies investigating a possible causal relationship between DM and *Candida*-related lesions. However, cross-sectional studies investigating the previously mentioned candidal manifestations showed a higher prevalence of median rhomboid glossitis (285), denture stomatitis (286, 287), and angular cheilitis (288) in patients with T1DM or T2DM, compared to healthy controls. In one study, the overall prevalence of *Candida*-related lesions was 15% in patients with DM, compared to 3% in healthy controls (285). One study found an increased overall prevalence of fungal infections—including candidiasis—in patients with DM (289), while another study in complete denture wearers did not find an increased prevalence (290). Furthermore, one study found increased counts of *Candida* pseudohyphae in patients with T1DM, compared to healthy controls (285). This might partly explain the increased prevalence of candidal infections the latter authors observed. However, other studies show conflicting results, as they did not find an increased colonization of *Candida* in individuals with T1DM and T2DM (291, 292).

#### Relationship With Diabetes Mellitus: Pathogenesis (*Candida*-Related Oral Lesions)

This section will discuss the pathogenesis of candidal lesions in relation to DM, again by means of the previously utilized pathologic phenomena related to DM: hyperglycemia, insulin resistance, dyslipidemia, hypertension, and immune dysfunction.

##### Hyperglycemia

Several studies found that the increased prevalence of *Candida*-related oral lesions was associated with poor metabolic control, indicating that hyperglycemia is important for this association (285, 293, 294). However, besides these observational studies, very little is known about its exact pathogenic role. There are no studies investigating the downstream effects of hyperglycemia that are involved in the well-known complications of DM (polyol, AGE/RAGE, PKC, and hexosamine pathways). Interestingly, one study showed that inhibition of AGE formation in diabetic rats improved the microbicidal activity of PMNs against *C. albicans* (295). However, it should be noted that this study concerned peritoneal PMNs and *Candida* infection, but AGEs might be involved in oral candidal lesions as well. This has not been confirmed or investigated yet.

As we have discussed before, salivary glucose levels are often increased in patients with DM due to hyperglycemia. Similar to the suggestions for cariogenic oral streptococci, glucose can also act as a source of nutrition for yeast species such as *Candida*. Indeed, several studies found an association between increased glucose levels in saliva and the growth of *Candida* in the oral cavity of patients with DM (292, 296).

##### Dyslipidemia

Two animal studies observed an increased systemic infection with *C. albicans* after inducing hyperlipidemia, with increased LDL or VLDL levels in particular (297, 298). It should be further elucidated whether this is the case for humans as well, and if this is also applicable specifically for the oral cavity.

##### Insulin resistance and hypertension

Currently, there are no studies that investigated the effect of insulin resistance or hypertension on *Candida* colonization or *Candida*-related oral lesions.

##### Immune dysfunction: impaired immune response and proinflammatory state

As we discussed before, patients with DM are susceptible for hyposalivation. Saliva has several innate immune defensive mechanisms to protect the oral mucosa against microorganisms such as *Candida*. Examples are mechanical washing, its buffering capacity and the presence of antifungal components such as histatins and mucins (299). If patients with DM develop hyposalivation, these mechanisms are impaired, and as such, hyposalivation is an important risk factor for oral candidiasis (300). This was confirmed in a study, where patients with both DM and hyposalivation had higher counts of *Candida* species in their saliva (247). One study showed that both hyposalivation and the presence of DM were independent predictors of oral candidiasis (299).

We discussed before that the impaired innate immune response makes patients with DM susceptible to infections. The decreased phagocytosis and killing capabilities of the PMNs might cause overgrowth of *Candida*, eventually causing candidal infection (301). Furthermore, we mentioned that patients with DM have increased adhesion of microorganisms to several cell types. Adhesion to epithelial cells of the oral cavity is a prerequisite for *Candida* colonization; interestingly, buccal and palatal epithelial cells from patients with DM showed increased adhesion capacities for *Candida* in *ex vivo* experiments (287, 302).

#### Relationship With Diabetes Mellitus (Non-candida Related Lesions)

Several studies investigated only one non-candida related mucosal lesions (303, 304), while others studied several types of mucosal lesions (289, 305–309), but none of those studies investigated possible underlying biologic mechanisms. For this reason, all individual lesion types that might be associated with DM are presented separately in Table 4, together with their clinical signs. This concerns: traumatic ulcer, actinic cheilitis, melanin pigmentation, fissured tongue, benign migratory glossitis (geographic tongue), leukoplakia, lichen planus, and other lichenoid lesions. The (pre)malignant mucosal lesions will be discussed in more detail in section Oral Cancer.

**Table 4:** Overview of non-candidal lesions that are possibly associated with diabetes mellitus.

**Lesion type**	**Clinical signs**	**Association with diabetes**
Traumatic ulcer	Damaged mucosa caused by mechanical, thermal, chemical, electrical or irradiation trauma.	Guggenheimer et al. (305) found an increased prevalence in patients with T1DM, but could not find an explanation for this in their data. Perhaps impaired wound healing seen in patients with DM might play a role.
Actinic cheilitis	Premalignant disorder of the lip characterized by inflammation, caused by exposure to sunlight.	Silva et al. (306) and de Souza Bastos et al. (289) found a high prevalence in patients with T1DM or T2DM, compared to controls, but their data could not explain this difference.
Melanin pigmentation	Pigmentation of oral mucosa with a broad range of clinical signs, caused by both exo- and endogenous factors.	Conflicting results, as some found an increased prevalence in patients with T1DM or T2DM (289), but others did not (307).
Fissured tongue	Deep grooves on the dorsal side of the tongue.	A few studies observe an increase in prevalence, and hypothesize that fissured tongue might be the result of aging and a dry mouth (289, 305, 307).
Benign migratory glossitis (geographic tongue)	An inflammatory condition of the dorsal mucosal surface of the tongue, with loss of lingual papillae as a result. This is visible as a red, smooth surface, with a shifting positioning.	Several studies reported a significant increase in the prevalence of geographic tongue in patients with T1DM or T2DM (288, 293). Guggenheimer et al. (305) also found more subjects with geographic tongue amongst patients with T1DM, but this difference was not significant.
Leukoplakia	Literally: white patch. It is a “predominantly white (premalignant) lesion of the oral mucosa that cannot be characterized as any other definable lesion.”	Studies investigating the relationship between leukoplakia and DM show contradictory results. Some found an association (308–310), whereas others did not (288, 289, 307).
Lichen planus and lichenoid lesions	An inflammatory condition with varying clinical appearances, affecting the skin, the oral mucosa, or both. It might cause white patches, red and swollen tissue, pain in varying degrees and a burning sensation. It can be considered as a premalignant disorder.	Again, there is no consensus in the literature, as some report an increased prevalence of lichen planus (303, 308) whereas others did not find a difference between patients with DM and healthy controls (304, 305, 307). As the name suggests, oral lichenoid lesions resemble oral lichen planus, but have a different pathogenesis, such as medication use, contact allergies and graft-vs.-host disease. In case of DM, medication use, such as hypoglycemic drug or anti-hypertensives, increases the risk for oral lichenoid lesions (311). Sometimes, this is referred to as “Grinspan syndrome” (312).

*T1DM, Diabetes Mellitus Type 1; T2DM, Diabetes Mellitus Type 2*.

#### Concluding Comments

From an epidemiologic point of view, the prevalence of candida-related oral mucosal lesions is increased in patients with DM. However, longitudinal studies are lacking, and not much is known about the pathogenesis behind the increased prevalence. It is highly likely that the impaired immune response enables *Candida* species to act as opportunistic organisms. Table 5 provides an overview of the literature that discusses the association between *Candida*-related lesions. As for non-candida related mucosal lesions, the evidence is too limited to establish a relationship with DM.

**Table 5:** Overview of studies investigating the association between diabetes mellitus and oral candidal lesions.

**Study**	**Study population**	**Study design**	**Relationship?**	**Authors' conclusions**
**EPIDEMIOLOGY**				
Bremenkamp et al. (291)	Group 1: Patients with T1DM (*n =* 39; 20 males, 19 females; aged 9–27 years). Group 2: Control group 1 (*n =* 50; age- and gender matched with group 1). Group 3: Patients with T2DM (*n =* 37; 8 males, 29 females; aged 37–78 years). Group 4: Control group 2 (*n =* 36 age- and gender matched with group 3).	Cross-sectional study	*No*	There were no differences in the frequency of *Candida* spp. between the diabetic group and the matched control groups.
Darwazeh et al. (292)	Group 1: Patients with DM (*n =* 41; 17 T1DM, 24 T2DM; mean age = 52 ± 16 years). Group 2: Control subjects without DM (*n =* 34; mean age = 52 ± 18 years).	Cross-sectional study	*No*	Patients with DM did not show higher *Candida* isolation compared to the controls. However, patients with DM and oral candidal colonization had significant higher levels of salivary glucose, compared to non-colonized patients.
de Lima et al. (290)	Group 1: Patients with T2DM (*n =* 30; 11 males, 19 females; mean age = 60 ± 9 years). Group 2: Healthy controls (*n =* 30; 9 males, 21 females; mean age = 63 ± 12 years).	Cross-sectional study	*No*	There were no significant differences in oral mucosal lesions, including *Candida*-related lesions, between patients with DM and healthy controls.
de Souza Bastos et al. (289)	Group 1: Patients with T2DM (*n =* 146; 56 males, 90 females; mean age = 53.10 ± 7.9 years). Group 2: Age- and gender matched controls (*n =* 111; 53 males, 58 females; mean age = 51.4 ± 10.3 years).	Cross-sectional study	*Yes*	A significantly higher prevalence of total fungal infections was observed in patients with DM, compared to the control subjects.
Dorocka-Bobkowska et al. (286)	Group 1: Patients with T2DM (*n =* 110, 47 males; 63 females; 63.2 ± 10.5 years). Group 2: Control subjects without DM (*n =* 50; 21 males, 29 females; mean age = 66.9 ± 8.8 years).	Cross-sectional study	*Yes*	The prevalence of denture stomatitis and its associated oral complaints, angular cheilitis, glossitis and oral dryness were significantly increased in patients with DM, compared to the controls.
Guggenheimer et al. (285)	Group 1: Patients with T1DM (*n =* 405; 204 males, 201 females; mean age = 33.0 ± 0.4). Group 2: Matched controls without DM (*n =* 268; 108 males, 160 females; mean age = 31.8 ± 0.49).	Cross-sectional study	*Yes*	The prevalence of several manifestations of candidal lesions (median rhomboid glossitis, denture stomatitis and atrophy of the tongue papilla) was increased in patients with DM, compared to controls.
Saini et al. (288)	Group 1: Patients with DM (*n =* 420; 29 T1DM, 309 T2DM; 185 males, 235 females; mean age = 53.0 ± 10.5 years). Group 2: Control subjects without DM (*n =* 420; 167 males, 253 females; mean age = 51.8 ± 11.6 years).	Cross-sectional study	*Yes*	The prevalence of denture stomatitis and angular cheilitis was increased in patients with DM, compared to the controls.
**PATHOGENESIS**				
**Hyperglycemia**				
Al Mubarak et al. (294)	Patients with T2DM (*n =* 42; 18 males, 24 females; mean age = 47.3 ± 14.4 years).	Cross-sectional study	*Yes*	Patients with poor metabolic control (HbA_1c_ >9%) had more candidal infections, compared to well-controlled patients (HbA_1c_ < 6%).
Al-Maweri et al. (293)	Group 1: Patients with T2DM (*n =* 391; 43.5% male, 56.5% female; mean age = 54.71 ± 8.48 years). Group 2: Control subjects without DM (*n =* 391; 38.9% males, 61.1% females; mean age = 53.04 ± 12.06 years).	Cross-sectional study	*Yes*	The prevalence of denture stomatitis and angular cheilitis was increased in patients with DM, compared to the controls. Within the group with DM, poor glycemic control was associated with increased prevalence of the same conditions.
Guggenheimer et al. (285)	Group 1: Patients with T1DM (*n =* 405; 204 males, 201 females; mean age = 33.0 ± 0.4). Group 2: Matched control subjects without DM (*n =* 268; 108 males, 160 females; mean age = 31.8 ± 0.49).	Cross-sectional study	*Yes*	The presence of *Candida* pseudohyphae was significantly increased in patients with DM, which was associated with glycemic control.
Sashikumar and Kannan (296)	Group 1: Well-controlled diabetic subjects (*n =* 50; aged 40–60 years). Group 2: Patients with poorly controlled T2DM (*n =* 50; age- and gender matched). Group 3: Age- and gender matched non-diabetic controls (*n =* 50).	Cross-sectional study	*Yes*	Colony-forming units of *Candida* were higher in patients with DM, and this was significantly correlated with levels of salivary glucose.
de Souza Ferreira et al. (295)	Group 1: Control rats, no AMG treatment (*n =* 8). Group 2: Control rats, + AMG treatment (*n =* 8). Group 3: Diabetic rats, no AMG treatment (*n =* 8). Group 4: Diabetic rats, + AMG treatment (*n =* 8). Rats used: Wistar rats (male, 280 ± 50 g).	Cross-sectional animal study	*Yes*	Microbicidal activity of the PMNs against *Candida* significantly increased in the rats that were treated with AMG, which inhibits the formation of AGEs. This implicates that there is a relationship between AGE formation and the host immune response against opportunistic infections. It should be noted that it concerned peritoneal PMNs and peritoneal *Candida* infection in this study.
**Dyslipidemia**				
Netea et al. (297)	Group 1: Low-density-lipoprotein-receptor-deficient mice (type: *Ldl–*/–). Group 2: Wild type control mice (type: 57BL/6J).	Longitudinal animal study	*Yes*	The *Ldlr* –/– mice developed 7–9 times higher LDL levels, compared to the control rats. These increased levels made them more susceptible for systemic *Candida* infection, which resulted in higher levels of inflammatory markers and a higher mortality rate.
Vonk et al. (298)	Group 1: Apolipoprotein E-deficient mice (*Apoe–/–*). Group 2: Weight matched control mice (*Apoe+/+*).	Cross-sectional animal study	*Yes*	VLDL levels were 8 times higher in *Apoe-/-* mice, compared to the matched controls. This resulted in higher mortality due to candidemia and higher levels of *C. Albicans* in plasma and kidney tissue. VLDL particles might serve as nutrition for *Candida*, and neutralize candidacidal properties of human serum.
**Immune dysfunction**				
Darwazeh et al. (302)	Group 1: Patients with DM (*n =* 50; 27 males, 23 females; mean age = 53.7 ± 14.5 years). Group 2: Control subjects without DM (*n =* 50; 23 males, 27 females; 56.3 ± 17.3 years).	Cross-sectional study and *ex-vivo* experiment	*Yes*	There was increased adhesion of *C. albicans* to the buccal mucosa of patients with DM, who also more frequently suffered from *Candida* infection.
Dorocka-Bobkowska et al. (287)	Group 1: T2DM denture-wearing patients (*n =* 70; 29 males, 41 females; mean age = 57 ± 8.8 years). Group 2: Denture-wearing control subjects without DM (*n =* 58; 27 males, 31 females; mean age = 58 ± 7.3 years)	Cross-sectional study and *ex-vivo* experiment	*Yes*	Not only did they found an increased prevalence of denture stomatitis in the diabetic group, but also an increased adherence of *C. albicans* to palatal epithelial cells. This indicates a predisposition for *Candida* infection in patients with DM.
Ueta et al. (301)	Group 1: Patients with symptomatic oral candidiasis and DM (*n =* 8). Group 2: Patients with symptomatic oral candidiasis, without DM (*n =* 64).	Cross-sectional study	*Yes*	White blood counts, levels of CRP and erythrocyte sedimentation rates were all increased in patients with DM and oral candidiasis. Oral PMNs in these patients produced less ROS and showed impaired phagocytosis and intracellular killing of *Candida* cells. This might indicate that the neutrophil suppression plays an important role in the predisposing state of DM for opportunistic infections.

*T1DM, Diabetes Mellitus Type 1; T2DM, Diabetes Mellitus Type 2; AMG, Aminoguanidine; PMN, Polymorphonuclear Neutrophil; VLDL, Very Low-Density Lipoprotein; CRP, C-reactive Protein*.

### Oral Cancer

#### Background

This section focuses on cancers of the lip, tongue, oral cavity, and oropharynx. Oral and oropharyngeal cancers together are ranked as the sixth most common type of cancer, but prevalence and incidence vary greatly between countries and regions (313). The global incidence was estimated to be 263,900 in 2008, causing 128,000 deaths worldwide (314). Many possible risk factors have been identified, including: smoking, tobacco and betel nut chewing, alcohol abuse, snuff dipping, sunlight, radiation, viruses, and immune dysfunction (313). Many risk factors are culture dependent, hence the varying epidemiologic figures across regions. For example, 75% of all cases in the US could be attributed to alcohol and tobacco use (315), while in Taiwan and neighboring countries, betel nut chewing was the main risk factor (314). Survival rates also vary between countries, age groups, and types of cancer, but in general, the 5-year survival is estimated to be ~50% for cancers of the tongue, oral cavity, and oropharynx, and 90% for cancers of the lip (313). As we mentioned in section Dry Mouth, radiation therapy of cancer in the head and neck region can cause dry mouth (229).

#### Relationship With Diabetes Mellitus: Epidemiology

Patients with DM have an increased risk for developing cancer in several organs and tissues, as well as an increase in cancer mortality, compared to subjects without DM (316). A recent meta-analysis showed that this is also the case for oral cancer (317). One cross-sectional Hungarian study showed an increased prevalence of oral cancer in patients with T2DM or T1DM compared to controls, as well as an over-representation of T2DM in individuals with oral cancer (310). A Brazilian cross-sectional study, which we also discussed in the previous section, revealed an increased prevalence of potentially malignant mucosal lesions (actinic cheilitis, lichen planus, leukoplakia, and nicotinic stomatitis) in patients with T2DM (289). A similar study from Malaysia showed contradicting results, as no association between DM and precancerous lesions was found (288). However, a large cross-sectional study from the US also observed that DM was an independent predictor for oral leukoplakia, which is considered as a pre-malignant lesion (318). The same was found in a cross-sectional study from India, but only for women (309). Two case-control studies from Italy presented contradicting results, as one did find an increased risk for cancer of the oral cavity in patients with DM (319), while the other did not (320).

There are also several longitudinal studies investigating the incidence of—and risk for—oral cancer in diabetic cohorts. One Danish population-based cohort study compared the incidence of several types of cancer in a large diabetic population (*n* = 109,581) with expected incidences based on a national cancer registry record. They found that the incidence of “cancer of the mouth” was increased 2-fold in the diabetic population younger than 50 years (321). Two large Taiwanese cohort studies from the same group also investigated the risk for head and neck cancer in patients with T2DM, with oral cancer specifically. The first did not find an increased risk, possibly due to a relatively short follow-up period (2 years), and no matching of study subjects (322). However, after matching the patients and prolonging the follow-up period in their second study, they showed an increased risk for oral cancer and oropharyngeal cancer in patients with T2DM (323).

Besides its effect on cancer incidence, DM also negatively affects the prognosis of patients with oral squamous cell carcinoma (OSCC). In another Taiwanese study, overall survival, recurrence-free survival, and cancer-specific survival were all decreased in patients with OSCC and DM, compared to patients with OSCC without DM (324). This was also observed in a large prospective cohort study from the US, where DM was associated with an increased risk for mortality from cancer of the oral cavity or pharynx (325).

#### Relationship With Diabetes Mellitus: Pathogenesis

The pathogenesis behind the epidemiologic association between DM an oral cancer is complex and remains to be fully elucidated. In an attempt to explain the increased risk for oral cancer by means of the pathological mechanisms that we have been using throughout this review, it appears that only hyperglycemia and dyslipidemia have been studied, albeit very limited. There is no data available on the role of hypertension, insulin resistance and immune dysfunction.

##### Hyperglycemia

Although it is likely that hyperglycemia is important for the increased risk of oral cancer in patients with DM, there is only limited evidence for that hypothesis. In one study, elevated levels of HbA_1c_ were associated with an increased risk for oral leukoplakia, a pre-malignant oral mucosal lesion (326). The downstream pathways of hyperglycemia (polyol, AGE/RAGE, PKC, hexosamine) are largely unexplored. Interestingly, inhibition of the polyol pathway in a diabetic animal model significantly lowered the number of premalignant lesions in the colon (327). It would be interesting to investigate whether this is the case for animal and human oral mucosal tissues as well.

One *in vitro* study observed increased cell migration when oral cancer cell lines were treated with AGEs. This was probably the result of increased expression of RAGE, MMP-2, and MMP-9 (328). Increased migration of cancer cells is one of the important characteristics of cancer malignancy. In another *in vitro* study, oral cancer cells were again treated with AGEs, which resulted in a decreased p53 expression. P53 is an important tumor suppressor, regulating cell survival and cell death; decreased expression usually promotes cell survival and inhibits cell death. Therefore, AGEs are probably involved in the survival rate of oral cancer cells through p53 suppression, and might worsen the prognosis of oral cancer in patients with DM (329). This poor prognosis was confirmed in patients with oral squamous cell carcinoma, where increased RAGE expression in tumors decreased disease-free survival (330). One study found that one specific RAGE gene polymorphism significantly increased the risk for oral cancer in patients with DM (331). RAGE also seems to be important for the invasiveness of oral mucosal tumor cells, since metastatic tumor cells showed a higher expression of RAGE. Suppression of RAGE in oral squamous carcinoma cell lines decreased the invasive activity (332).

##### Dyslipidemia

One study had a closer look at the individual components of the metabolic syndrome (hyperglycemia, elevated triglycerides, lowered HDL levels, hypertension, and central obesity), which revealed that subjects with hypertriglyceridemia had a higher risk for developing oral pre-malignancies (333).

#### Concluding Comments

Table 6 presents an overview of the literature that discusses the association between DM and oral cancer. As is the case for other types of cancer, epidemiologic research seems to indicate that the prevalence and even incidence of oral premalignant lesions and oral cancer is increased in patients with DM. Considering the potentially devastating and even fatal effects of oral cancer, these findings are worrying. There is a lack of research into possible pathogenic pathways that explain the association between DM and oral cancer. As long as it remains unknown why patients with DM are at higher risk for oral cancer, targeted prevention and treatment programs are unlikely to succeed.

**Table 6:** Overview of studies investigating the association between diabetes mellitus and oral cancer.

**Study**	**Study population**	**Study design**	**Relationship?**	**Authors' conclusions**
**EPIDEMIOLOGY**				
Bosetti et al. (319)	Group 1: Patients with cancer of the oral cavity/pharynx (*n =* 1,468; 1,190 males, 278 females; median age = 58, self-reported DM: *n =* 98). Group 2: Cancer-free controls (*n =* 3,761; 2553 males, 1,208 female; median age = 58, self-reported DM *n =* 181).	Integrated case-control studies	*Yes*	The risk for oral and pharyngeal cancer was increased in patients with DM, compared to the group without DM (OR = 1.58, 95% CI = 1.15–2.18), after adjustment for gender, age, study center, year of interview, education, alcohol drinking, tobacco smoking, and BMI.
Campbell et al. (325)	Subjects free of cancer at baseline (*n =* 1,053,831), followed from 1982 to 2008.	Prospective cohort study	*Yes*	After multivariable adjustment, diabetes was associated with an increased mortality from cancer of the oral cavity and pharynx in men (RR = 1.44, 95% CI: 1.07–1.94).
de Souza Bastos et al. (289)	Group 1: Patients with T2DM (*n =* 146; 56 males, 90 females; mean age = 53.10 ± 7.9 years). Group 2: Age- and gender matched controls (*n =* 111; 53 males, 58 females; mean age = 51.4 ± 10.3 years).	Cross-sectional study	*Yes*	The prevalence of potentially malignant disorders (actinic cheilitis, lichen planus, leukoplakia and nicotinic stomatitis) was significantly increased in patients with T2DM.
Dietrich et al. (318)	Group 1: Patients with oral leukoplakia (*n =* 65; 12 with DM, 53 without DM; 69.2% male, 30.8% female; mean age = 57.5 years). Group 2: Controls (*n =* 15,746; 1,289 with DM, 14,457 without DM; 47.4% male, 52.6% female; mean age = 47.9 years).	Cross-sectional study	*Yes*	Oral leukoplakia was more prevalent in patients with DM (0.92%), compared to patients without DM (0.37%). Besides smoking, age, and socio-economic status, DM was found to be an independent predictor for the development of oral leukoplakia (OL). Patients with DM were 3 times more likely to develop OL (weighed OR = 3.03, 95% CI: 1.28–7.21).
Dikshit et al. (309)	Group 1: Patients with leukoplakia (*n =* 927) or erythroplakia (*n =* 100). Group 2: Healthy controls (*n =* 47,773).	Case-control study	*Yes*	In women, DM was an independent risk factor for oral leukoplakia (OR = 2.0, 95% CI: 1.4–2.9) and erythroplakia (OR = 3.2, 95% CI: 1.3–7.9). For men, no such association was found.
Gong et al. (317)	13 epidemiological studies on the association between oral cancer and DM (4 case-control and 9 cohort studies, combined *n* >4.8 million; 6,456 patients with oral cancer). 4 case-control studies on the association between oral precancerous lesions and DM (1,407 patients with oral precancerous lesions).	Systematic review with meta-analysis	*Yes*	Analysis of 4 case-control studies and 9 cohort studies revealed a significant association between oral cancer (SRR = 1.15, 95% CI: 1.02–1.29) and oral cancer related mortality (SRR = 1.41, 95% CI: 1.16–1.72) and T2DM. Meta-analysis of the case-control study revealed a positive association between T2DM and the risk for precancerous lesions (SRR = 1.85, 95% CI: 1.23–2.80).
La Vecchia et al. (320)	Group 1: Patients with histologically confirmed incident cancer (*n =* 9,991). Group 2: Healthy control subjects (*n =* 7.834).	Integrated case-control studies	*No*	There was no significant increase in the risk for cancer of the oral cavity (RR = 0.50, 95% CI: 0.2–1.1) in patients with DM.
Saini et al. (288)	Group 1: Patients with DM (*n =* 420; 29 T1DM, 309 T2DM; 185 males, 235 females; mean age = 53.0 ± 10.5 years). Group 2: Control subjects without DM (*n =* 420; 167 males, 253 females; mean age = 51.8 ± 11.6 years).	Cross-sectional study	*No*	There was no association between the presence of DM and the prevalence of oral precancerous lesions.
Tseng (322)	Group 1: Patients with T2DM (*n =* 115,692; 45.3%, male, 54.7% female). Group 2: Patients without T2DM (*n =* 882,849; 50.1 males, 49.9% female).	Retrospective cohort study	*No*	After multivariable adjustment, there was no significant association between T2DM and incidence oral cancer after 2-year follow-up (RR = 1.195, 95% CI: 0.892–1.601).
Tseng et al. (323)	Group 1: Patients with DM (*n =* 89,089; 52.9% male, 47.1% female; mean age = 55.4 ± 15.1 years). Group 2: Matched patients without DM (*n =* 89,089; 52.9% male, 47.1% female; mean age = 55.4 ± 15.1 years).	Retrospective cohort study	*Yes*	The risk for developing oral cancer and oropharyngeal cancer was significantly higher in patients with DM, compared to the matched controls (adjusted HR = 1.74, 95% CI 1.47–2.06 for oral cancer and 1.53, 95% CI: 1.01–2.31 for oropharyngeal cancer).
Ujpál et al. (310)	*First analysis:* Group 1: Patients with DM (*n =* 200; 82 T1DM, 118 T2DM; 69 males, 131 females; mean age = 45.8 years). Group 2: Control subjects without DM (*n =* 280; 109 males, 171 females; mean age = 47.2 years). *Second analysis:* Group 1: Patients with malignant oral tumors (*n =* 610; 435 males, 175 females; mean age = 56 years). Group 2: Tumor- and complaint free controls (*n =* 574; 351 males, 223 females; mean age = 51 years).	Cross-sectional study	*Yes*	The first cross-sectional analysis showed an increased prevalence of benign tissue accumulations (14.5%) and precancerous lesions (8%) in patients with T1DM or T2DM, compared to control subjects without DM (6.4% and 3.2% respectively). The second cross-sectional analysis showed an increased prevalence of diabetes in the oral cancer patient group, compared to the cancer-free controls.
Wideroff et al. (321)	Group 1: Patients diagnosed with diabetes (*n =* 109,581). Group 2: National cancer registry records.	Population-based cohort study	*Yes*	The observed numbers of incident cancers of the mouth in the diabetic population were compared with the expected incidence, based on the national cancer registry records. Even after adjustment for multiple variables, the relative incidence ratios remained significantly increased (1.8, 95% CI: 1.2–2.6).
Wu et al. (324)	Group 1: Patients with OSCC and DM (*n =* 71; 62 males, 9 females; mean age = 58.9 ± 12.4 years). Group 2: Patients with OSCC, without DM (*n =* 301; 270 males, 31 females; mean age = 51.2 ± 12.6 years).	Retrospective cohort study	*Yes*	OSCC patients with DM showed a decreased overall survival (HR = 2.22, 95% CI: 1.27–3.88), recurrence-free survival (HR = 2.42, 95% CI: 1.49–3.92), and cancer-specific survival (HR = 2.16, 95% CI: 1.17–3.97), compared to the OSCC patients without DM, even after multivariable adjustment.
**PATHOGENESIS**				
**Hyperglycemia**				
Meisel et al. (326)	Group 1: Patients with leukoplakia (*n =* 123; 68 males, 55 females; mean age = 55.2 ± 15.5 years). Group 2: Patients without leukoplakia (*n =* 246; 136 males, 110 females; mean age = 55.2 ± 15.6 years).	Cross-sectional study	*Yes*	Conditional regression analysis revealed a higher probability for leukoplakia with increasing HbA_1c_ levels (OR = 1.51, 95% CI: 1.08–2.12). When correcting for the interaction with smoking status, the OR for never-smokers was 1.96 (95% CI: 1.15–3.37), and for ever-smokers 1.29 (95% CI: 0.80–2.09).
*Advanced glycation endproducts*				
Bhawal et al. (332)	*Analysis 1:* Group 1: Oral primary tumors and tumor-containing metastatic lymph nodes. Group 2: The corresponding normal oral mucosa. *Analysis 2:* Oral squamous cell carcinoma cell lines.	*In vitro* experiment	*Yes*	The number of cells expressing RAGE was significantly higher in metastatic tumors, compared to normal mucosal cells. Also, suppressing the expression of RAGE in oral squamous cell carcinoma cell lines resulted in a decreased invasive activity and absolute number of invasive cells. This suggests that RAGE expression is involved in the invasiveness of oral malignant tumors.
Ko et al. (328)	Oral cancer cell lines (SAS), treated with AGEs or BSA (negative control).	*In vitro* experiment	*Yes*	Increased cell migration was observed when the oral cancer cells were treated with AGEs. This was probably the result of increased expression of RAGE, MMP-2 and MMP-9. The authors suggest that this increases the invasiveness of oral cancer cells, since migration is an important feature of malignancy.
Ko et al. (329)	Oral cancer cell lines (SAS), treated with AGEs or BSA (negative control).	*In vitro* experiment	*Yes*	The oral cancer cells that were treated with AGEs, showed a decreased p53 expression. A decreased expression of p53 usually promotes cell survival and suppresses cell death. The authors therefore conclude that AGEs are probably involved in the survival rate of oral cancer cells, which might worsen the prognosis of oral cancer in patients with DM.
Sasahira et al. (330)	Group 1: Patients with OSCC and high expression of RAGE (*n =* 30). Group 2: Patients with OSCC without high expression of RAGE (*n =* 44).	Prospective cohort study	*Yes*	High expression of RAGE in OSCCs was associated with the depth of invasion and local recurrence of the cancer. Disease-free survival was negatively impaired by high expression of RAGE.
Su et al. (331)	Group 1: Patients with oral cancer (*n =* 618; 96.4% male, 3.6% female; mean age = 54.3 ± 11.3) Group 2: Cancer-free control subjects (*n =* 592; 81.9% male, 18.1% female; mean age = 51 ± 15).	Cross-sectional study	*Yes*	Patients with a specific RAGE polymorphism had an increased risk for oral cancer (aOR = 2.053, 95% CI: 1.269–3.345), even after correction for multiple confounders (age, gender, betel nut chewing, and tobacco consumption).
*Polyol pathway*				
Saxena et al. (327)	Group 1: Control diabetic mice (*n =* 6). Group 2: Diabetic mice with AOM-induced colon carcinogenesis (*n =* 6). Group 3: Diabetic mice with AOM-induced colon carcinogenesis, treated with a polyol pathway inhibitor (*n =* 6). C57BL/KsJ-db/db obese mice were used.	Longitudinal animal study	*Yes*	By inhibiting the polyol pathway, significantly fewer premalignant lesions were observed in the colons of diabetic mice. This indicates that the polyol pathway is involved in the development of diabetes-associated colon cancer. Perhaps the same accounts for oral cancer as well.
**Dyslipidemia**				
Meisel et al. (326)	Group 1: Patients with leukoplakia (*n =* 123; 68 males, 55 females; mean age = 55.2 ± 15.5 years). Group 2: Patients without leukoplakia (*n =* 246; 136 males, 110 females; mean age = 55.2 ± 15.6 years).	Case-control study	*Yes*	Besides HbA_1c_, LDL and total cholesterol levels were also increased in patients with leukoplakia. The conditional regression analysis for LDL-cholesterol revealed an OR of 3.01 in “never smokers” and an OR of 1.47 in “eversmokers.”
Yen et al. (333)	Group 1: Patients with metabolic syndrome (*n =* 17,206) Group 2: Patients without metabolic syndrome (*n =* 60,564)	Cross-sectional study	*Yes*	The prevalence of at least one oral premalignant lesion was significantly higher in the group with metabolic syndrome, compared to the control group. Multivariate logistic regression analysis revealed that hyperglycemia (aOR = 1.30, 95% CI: 1.02–1.67) and hypertriglyceridemia (aOR = 1.43, 95% CI: 1.17–1.75) independently increased the risk for an oral premalignant lesion.

*T1DM, Diabetes Mellitus Type 1; T2DM, Diabetes Mellitus Type 2; BMI, Body Mass Index; OL, Oral Leukoplakia; SRR, Standardized Relative Risk; OSCC, Oral Squamous Cell Carcinoma; (R)AGE, (Receptor for) Advanced Glycation Endproducts; MMP, Matrix Metalloproteinase; LDL, Low Density Lipoprotein*.

### Taste Disturbance

#### Background

Gustatory dysfunction (i.e., taste disturbance) is generally classified into three major types: *dysgeusia*, a distortion or alteration in taste sensation; *hypogeusia*, a partial loss of taste and *ageusia*, a complete loss of taste (334). Compared to for example olfactory dysfunction, gustatory dysfunction is relatively rare. Estimations of the prevalence of any disturbance of taste in the adult population range from 0.93% in the US (335) to 2.5% in Sweden (336). The prevalence of severe hypogeusia or ageusia is lower, with 0.83% of a study population that attended a chemosensory clinic. In the general population, this will probably be even lower (337). As is the case for other sensory functions, such as hearing or smell, the risk for gustatory dysfunction increases with age (338). The prevalence is doubled in individuals aged 45–65, and even tripled in those aged 66 and older, compared to subjects aged 18–44 (335). Especially women aged 50 years or older seem to be susceptible for taste disturbances (336). Besides older age, many other risk factors have been proposed in literature, including physiological changes, oral and systemic diseases, iatrogenic causes, nutritional deficiencies, and lifestyle behavior (338).

#### Relationship With Diabetes Mellitus: Epidemiology

Epidemiologic research into the association between DM and taste disturbances is limited to a few cross-sectional studies. Nevertheless, both hypogeusia and ageusia were observed more frequently in patients with T1DM (339, 340) and T2DM (340, 341), compared to healthy controls. Another way of defining taste disturbances is by measuring detection thresholds for certain tastes (e.g., sweet or salty), after exposure to certain taste stimuli (e.g., chemical or electrical), with higher thresholds indicating reduced taste sensation. In both patients with T1DM (339, 340, 342) and patients with T2DM (340, 343–345), these thresholds were generally elevated, compared to healthy controls. One study did not find an association (346). Taste disturbances can have important consequences, especially for patients with DM. Research has shown that diminished taste perception resulted in a tendency toward consumption of beverages with a higher sucrose concentration with a higher sweet taste intensity. This might indicate a preference for sweeter foods or beverages that probably are higher in carbohydrates and calories (347). Besides a possible increase in glucose supplies, this could also increase the risk to develop obesity, a major risk factor for T2DM and its complications.

#### Relationship With Diabetes Mellitus: Pathogenesis

Unfortunately, there is no literature available that investigated the same pathogenic pathways we have been using throughout this report. However, a few explanations why patients with DM more frequently suffer from taste impairment have been proposed. For example, the increased susceptibility for dry mouth we discussed in section Dry Mouth, might increase the risk for taste disturbances. One study found that, of all patients with a subjective complaint of taste disturbance, 63% also had a sensation of oral dryness, often regardless of the actual salivary flow (336). Another study showed that hyposalivation was indeed associated with hypogeusia in a general elderly population (348). Furthermore, since gustation is a sensory function with neural involvement, it is conceivable that the typical neuropathy associated with DM is one of the causative factors for impaired gustatory function in patients with DM. Three studies from the same research group showed that taste impairment in individuals with T1DM was significantly associated with peripheral neuropathy (339, 349, 350). One animal study observed decreased innervation of taste buds in diabetic rats, compared to non-diabetic control rats (351). Another study with diabetic rats showed increased cell apoptosis in their taste buds (352). Humans with T1DM also had decreased vascularization of the tip of the tongue (353). The above findings indicate that the tongue, with the taste buds in particular, is susceptible for vascular and neuronal damage.

#### Concluding Comments

Studies investigating the association between taste impairments and DM can be found in Table 7. Although it remains a relatively rare condition, patients with DM seem to be susceptible for certain forms and gradations of taste impairment. However, there is a lack of solid longitudinal research, and many common risk factors can obscure a possible association. Besides a few careful suggestions that neural and vascular degeneration might play a role, the pathogenesis remains largely unexplored.

**Table 7:** Overview of studies investigating the association between diabetes mellitus and taste impairment.

**Study**	**Study population**	**Study design**	**Relationship?**	**Authors' conclusions**
**EPIDEMIOLOGY**				
De Carli et al. (345)	Group 1: Patients with T2DM (*n =* 25; 72% male, 28% female; mean age = 56.8 ± 6.7 years). Group 2: Healthy controls (*n =* 25; 72% male, 28 female; mean age = 56.2 ± 4.9 years).	Cross-sectional study	*Yes*	Thresholds for sweet, salt, sour, and bitter taste recognition were increased in patients with T2DM, compared to the controls.
Gondivkar et al. (341)	Group 1: Patients with well controlled T2DM (*n =* 40; 21 males, 19 females; mean age = 40.97 ± 5.35 years). Group 2: Patients with poorly controlled T2DM (*n =* 40; 25 males, 15 females; mean age = 43.52 ± 6.32 years). Group 3: Control subjects without DM (*n =* 40; 23 males, 17 females; mean age = 40.10 ± 5.18 years).	Cross-sectional study	*Yes*	Patients with either well or poorly controlled diabetes suffered from hypogeusia and ageusia more often, compared to healthy controls. Especially sweet taste was impaired, followed by sour and salt. They hypothesize that the impaired taste for sweet might result in increased consumption of sweet food, and beverages, (mostly high in sugar) worsening the hyperglycemia.
Khobragade et al. (342)	Group 1: Patients with T1DM (*n =* 70; 38 males, 32 females; age range 20–45 years). Group 2: Age and weight matched control subjects without DM (*n =* 70; 40 males, 30 females).	Cross-sectional study	*Yes*	The taste thresholds for sweet, salt, sour and bitter was significantly increased in the patients with DM, which indicates an impaired taste sensation.
Le Floch et al. (339)	Group 1: Patients with T1DM (*n =* 57; 29 males, 28 females; mean age = 40.9 ± 2.0 years). Group 2: Control subjects without DM (*n =* 38; 17 males, 21 females; mean age = 45.5 ± 1.6 years).	Cross-sectional study	*Yes*	Hypogeusia was observed in significantly more patients with DM than in control subjects without DM. This concerned all four primary tastes, and the taste impairment was significantly associated with other diabetic complications. The association with peripheral neuropathy was the strongest.
Naka et al. (346)	Group 1: Patients with uncomplicated DM (*n =* 29; 20 males, 9 females; mean age = 46.3 ± 15.7 years). Group 2: Patients with DM and vascular complications (*n =* 24; 9 males, 15 females; mean age = 57.9 ± 15.6 years). Group 3: Patients with DM and complicating diseases (*n =* 23; 12 males, 11 females; mean age = 54.7 ± 16.0 years). Group 4: Control subjects without DM (*n =* 29; 20 males, 9 females; mean age = 45.6 ± 15.3 years).	Cross-sectional study	*No*	There was no significant difference in gustatory function (for the four basic tastes) between patients with DM and control subjects without DM, assessed by using impregnated taste strips.
Perros et al. (343)	Group 1: Patients with newly diagnosed T2DM (*n =* 20). Group 2: Age-, BMI- and gender matched control subjects without DM (*n =* 20). Group 3: Patients with DM (*n =* 11; 5 T1DM, 6 T2DM), with a disease duration of >10 years.	Cross-sectional study	*Yes*	The patients with newly-diagnosed diabetes had increased EGT, detection threshold for glucose, and recognition threshold for glucose and salt. This might indicate a preference for sweet nutrients, increasing the risk for elevated blood glucose levels.
Stolbova et al. (340)	Group 1: Patients with T2DM (*n =* 73; 26 males, 47 females; mean age = 57.7 ± 14.0 years). Group 2: Patients with T1DM (*n =* 11; 4 males, 7 females; mean age = 47.6 ± 16.1 years). Group 3: Obese control subjects without DM (*n =* 12; 4 males, 8 females; mean age = 49.7 ± 12.0 years). Group 4: Control subjects (*n =* 29; mean age = 25.6 ± 9.5 years).	Cross-sectional study	*Yes*	The gustometric threshold was significantly higher in the diabetic and obese subject, compared to the controls. Also, hypogeusia and ageusia were observed in patients with T1DM or T2DM, as well as in obese patients, but not in the control subjects.
Wasalathanthri et al. (344)	Group 1: Control subjects without DM (*n =* 34; 11 males, 23 females; mean age = 45.1 ± 8.9 years). Group 2: Patients with pre-diabetes (*n =* 40; 17 males, 23 females; mean age = 45.9 ± 9.4 years). Group 3: Patients with T2DM (*n =* 40; 22 males, 18 females; 45.7 ± 8.4 years).	Cross-sectional study	*Yes*	The patients with T2DM had impaired sweet taste sensitivity, compared to the controls. Furthermore, even though the taste sensitivity of the patients with pre-diabetes lay in between those of patients with DM and controls, this could not be statistically proven.
**PATHOGENESIS**				
Cheng et al. (352)	Group 1: Diabetic rats (induced with high-fat diet and streptozotocin injection). Group 2: Non-diabetic rats Rats used: male, Wistar, 8–10 weeks old, 180–200 g.	Cross-sectional animal study	*Yes*	The diabetic rats showed increased cell apoptosis of the taste buds, compared to the non-diabetic rats. Further analysis seems to hint that the apoptosis of the cells in the taste buds is mediated by the intrinsic mitochondrial pathway through increased BCL2 and decreased BAX expression.
Le Floch et al. (350)	Group 1: Patients with DM (*n =* 73). Group 2: Age- and gender matched control subjects without DM (*n =* 25).	Prospective cohort study	*Yes*	The EGT, which is an indicator for taste loss, significantly increased in both groups, but was higher in the patients with DM. Moreover, this increase was significantly associated with other degenerative complications, especially neuropathy, which indicates a similar biologic mechanism.
Le Floch et al. (349)	Group 1: Patients with T1DM (*n =* 50; 26 males, 24 females; mean age = 29.1 ± 3.3 years). Group 2: Age- and gender matched control subjects without DM (*n =* 50; 26 males, 24 females; 29.2 ± 3.3).	Cross-sectional study	*Yes*	The EGT was significantly higher in the diabetic group, compared to the control subjects without DM. The increased EGT was significantly associated with diabetic peripheral neuropathy and nephropathy, suggesting that taste impairment is another degenerative complication of DM.
Pai et al. (351)	Group 1: Diabetic rats (induced by streptozotocin injection). Group 2: Non-diabetic control rats. rats used: adult, male, Sprague-Dawley rats, weighing 300–350 g.	Cross-sectional animal study	*Yes*	The number of nerve fibers in the papillae and taste cells in the taste buds were decreased in the diabetic rats after 20 weeks of streptozotocin injection. After this period, the diabetic rats also had fewer numbers of these fibers and cells, compared to the control rats. This might indicate that taste impaired in patients with DM is caused by neuropathic damage and/or morphological changes in the taste buds
Pavlidis et al. (353)	Group 1: Patients with T1DM (*n =* 14; 6 males, 8 females; mean age = 48 ± 3.2 years) and patients with T2DM (*n =* 22; 7 males; 15 females; mean age = 46 ± 2.6 years). Group 2: Control subjects without DM (*n =* 36; 13 males, 23 females; mean age = 47 ± 2.3 years).	Cross-sectional study	*Yes*	Patients with DM had increased taste thresholds, measured by EGM. Furthermore, differences in gustatory anatomical structures were observed between the two groups. The patients with DM had a decreased density of fungiform papillae and worsened vascularization of the tongue tip, measured by contact endoscopy.

*T1DM, Diabetes Mellitus Type 1; T2DM, Diabetes Mellitus Type 2; EGT, Electrogustometric Threshold; BCL2, B-cell lymphoma 2; BAX, Bcl-2-Associated X Protein; STZ, Streptozotocin; EGM, Electrogustometry*.

### Other Oral Complications

The following conditions affecting the oral cavity have been mentioned in the literature as possible complications of DM, but evidence for an epidemiologic or pathologic association is too scarce to dedicate a separate section. Table 8 provides an overview of the literature that discusses the associations between each of these oral conditions and DM.

**Table 8:** Overview of studies investigating the association between diabetes mellitus and other oral complications.

**Study**	**Study population**	**Study design**	**Relationship?**	**Authors' conclusions**
**TEMPOROMANDIBULAR JOINT DYSFUNCTION**				
Collin et al. (354)	Group 1: Patients with T2DM (*n =* 45; 32 males, 13 females; mean age = 68 ± 5.9 years). Group 2: Control subjects without DM (*n =* 77; 32 males, 45 females; mean age = 67 ± 5.2 years).	Cross-sectional study	*Yes*	Severe temporomandibular joint (TMJ) dysfunction was significantly more prevalent in the diabetic group (27.3%), compared to the control group (15.8%). Also, peripheral diabetic neuropathy emerged as independent risk factor for TMJ dysfunction.
Uemura et al. (355)	Group 1: Diabetic rats (*n =* 10; diabetes induced by streptozotocin (STZ) injection). Group 2: Non-diabetic control rats (*n =* 10). Rats used in both groups: adult, male, Wistar rats (*n =* 5) and adult, male Goto-Kakizaki (GK) rats (*n =* 5]).	Cross-sectional animal study	*Yes*	The thickness of the articular disk of the TMJ was significantly lower in the diabetic rats, compared to the healthy controls. Also, in the retrodiscal tissue, the diameter of the capillaries was significantly decreased, possibly indicating microangiopathy.
**BURNING MOUTH SYNDROME**				
Collin et al. (354)	Group 1: Patients with T2DM (*n =* 45; 32 males, 13 females; mean age = 68 ± 5.9 years). Group 2: Control subjects without DM (*n =* 77; 32 males, 45 females; mean age = 67 ± 5.2 years).	Cross-sectional study	*Yes*	The prevalence of glossodynia (burning sensation of the tongue) was significantly higher in the diabetic group (18%) than in the control group (7%).
Eltas et al. (356)	Group 1: Patients with DM (*n =* 22; 41% male, 59% female; mean age = 44.6 years). Group 2: Control subjects without DM (*n =* 27; 44% male, 56% female; mean age = 40.4 years).	Cross-sectional study	*Yes*	BMS was significantly more prevalent in the diabetic group (40.9%), compared to the control group (11.1%).
Moore et al. (357)	Group 1: Patients with T1DM (*n =* 371; 50.4% male, 49.7% female; mean age = 33.4 ± 0.4 years). Group 2: Control subjects without DM (*n =* 233; 37.3% male, 62.7% female; mean age = 33.1 ± 0.5 years).	Cross-sectional study	*Yes*	The prevalence of BMS without any apparent pathology did not differ between the groups. However, within the diabetic group, BMS was significantly associated with diabetic peripheral neuropathy.
Vesterinen et al. (358)	Group 1: Patients with DM and chronic kidney disease (*n =* 51; 37 males, 14 females; mean age = 52 ± 13.5 years). Group 2: Patients with only chronic kidney disease (*n =* 87; 57 males, 30 females; mean age = 54 ± 12.6 years).	Cross-sectional study	*No*	There were no statistical significant differences in the prevalence of BMS between the patients with chronic kidney disease and diabetes, compared to the control patients with only chronic kidney disease.
**PULP NECROSIS AND APICAL PERIODONTITIS**				
Arya et al. (359)	Group 1: Patients with T2DM (*n =* 21; 12 males, 9 females; mean age = 51 years). Group 2: Patients without DM (*n =* 25; 10 males, 15 females; mean age = 34).	Prospective cohort study	*Yes*	Periapical healing after endodontic treatment was significantly lower in patients with DM (43%), compared to the group without DM (80%).
Catanzaro et al. (360)	Group 1: Healthy control rats (*n =* 8). Group 2: 30-days diabetic rats (*n =* 10, induced by streptozotocin injection) Group 3: 90-days diabetic rats (*n =* 10, induced by streptozotocin injection) Rats used: male, Wistar, 200–250 g.	Longitudinal animal study	*Yes*	Several inflammatory markers were elevated in the pulp of diabetic rats compared to the controls, such as nitrite concentrations, kallikrein and myeloperoxidase activity and alkaline phosphatase concentration. Collagen concentration was decreased in diabetic rats.
Fouad and Burleson (361)	Group 1: Patients with T1DM (*n =* 58) and T2DM (*n =* 184). Group 2: Control subjects without DM (*n =* 5,002).	Prospective cohort study	*Yes*	Of the included subjects, 540 non-surgically treated endodontic patients (of which 73 with DM) completed follow-up. Multivariate analysis revealed that a history of DM in patients with preoperative periradicular lesions (indicating endodontic infection) decreased the success of endodontic treatment.
Garber et al. (362)	Group 1: Healthy control rats (*n =* 11). Group 2: Diabetic rats (*n =* 11, induced by streptozotocin injection)	Cross-sectional animal study	*Yes*	Inflammation was observed significantly more often in dental pulp of diabetic rats, compared to controls. Also, the formation of dentin bridges, which indicates healing, was impaired more often in diabetic rats.
Lopez-Lopez et al. (363)	Group 1: Patients with T2DM (*n =* 50; 20 males, 30 females; mean age = 60.7 ± 10.3 years). Group 2: Healthy controls (*n =* 50; 22 males, 28 females; mean age = 61.6 ± 10.4 years).	Cross-sectional study	*Yes*	AP was more prevalent in patient with T2DM (74%) than in the control group (42%, *p* = 0.002). Also, after multivariate regression analysis, adjusting for several confounders, the association between AP (OR = 3.3; 95% CI: 1.4–8.0) and the number of root-filled teeth (OR = 1.7; 95% CI: 1.2–2.4) and DM remained significant.
Marotta et al. (364)	Group 1: Patients with T2DM (*n =* 30; 12 males, 18 females; mean age = 58.2 ± 8.2 years). Group 2: Age- and gender matched controls (*n =* 60; mean age = 58.3 ± 8.0 years).	Cross-sectional study	*Yes*	Patients with DM had significantly more teeth affected by AP (15%), compared to the control subjects (12%, p = 0.05).
Nakajima et al. (365)	*Experiment 1* Group 1: Diabetic rats (*n =* 7, rats used: Otsuka Long-Evans Tokushima Fatty Rats). Group 2: Control rats (*n =* 7, rats used: age-matched Long-Evans Tokushima Otsuka rats). *Experiment 2* Dental pulp cells from 8-week-old male Wistar rats were derived and cultured.	*Experiment 1:* cross-sectional animal study *Experiment 2: Ex vivo* experiment	*Yes*	Expression of RAGE and several inflammatory pathway markers (S100A8, S100A9, and IL-1β) were increased in pulp of diabetic rats, compared to the control rats. The dental pulp cell lines were treated with AGE, which resulted in increased expression of the same inflammatory pathways.
Sanchez-Dominguez et al. (366)	Group 1: Patients with poorly regulated T2DM (*n =* 59; 29 males, 30 females; mean age = 65.5 ± 10.6 years; HbA_1c_ ≥6.5%). Group 2: Patients with well-regulated T2DM (*n =* 24; 12 males, 12 females; 67.2 ± 10.8 years; HbA_1c_ < 6.5%).	Cross-sectional study	*Yes*	In a multivariate logistic regression analysis, the presence of AP in at least 1 tooth was significantly associated with poor metabolic control (HbA_1c_ ≥6.5%).
Segura-Egea et al. (367)	Group 1: Patients with T2DM (*n =* 32; 12 males, 20 females; mean age = 63.1 ± 8.3 years). Group 2: Control subjects without DM (*n =* 38; 16 males, 22 females; mean age = 59.6 ± 7.4 years).	Cross-sectional study	*Yes*	The prevalence of AP in at least one teeth was higher in the diabetic group 81.3%), compared to the control group (58%, p = 0.040). Also, in patients with DM, 7% of the teeth was affected by AP, compared to 4% in the control group (*p* = 0.007).
Segura-Egea et al. (368)	7 epidemiological studies, including patients with root canal treatment (total *n =* 1,593; 582 patient with DM, 1,011 control subjects without DM).	Systematic review and meta-analysis	*Yes*	In patients with DM, the prevalence of periapical radiolucent lesions was significantly increased, compared to the control subjects (OR = 1.42; 95% CL: 1.11–1.80; *p* = 0.006)
Smadi (369)	Group 1: Patients with T2DM (*n =* 145; 82 well-controlled T2DM [HbA_1c_ < 7%], 63 poor-controlled [HbA_1c_ >7%]; 49% male, 51% female; mean age = 49.6 ± 8.9 years) Group 2: Patients without DM (*n =* 146; 51% male, 49% female; mean age = 52.0 ± 9.0 years).	Cross-sectional study	*Yes*	The prevalence of teeth with AP and teeth with endodontic treatment were both increased in patients with T2DM. Patients with poorly controlled T2DM had a higher prevalence of AP and teeth with endodontic treatment, compared to well-controlled subjects.
**PERI-IMPLANTITIS**				
Al-Sowygh et al. (370)	Group 1: Patients with HbA_1c_ 6.1–8% (*n =* 25; 13 males, 12 females; mean age = 51.5 years). Group 2: Patients with HbA_1c_ 8.1–10% (*n =* 25; 15 males, 10 females; mean age = 53.7 years). Group 3: Patients with HbA_1c_ >10% (*n =* 17; 10 males, 7 females; mean age = 55.9 years). Group 4: Control subjects without DM with HbA_1c_ < 6% (*n =* 26; 13 males, 13 females; mean age = 50.1 years).	Cross-sectional study	*Yes*	Plaque index, bleeding on probing, pocket depth and jaw bone loss were all increased in the diabetic groups, compared to the control group. Especially the poorly controlled groups (2 and 3) showed higher values. AGEs measured in peri-implant sulcular fluid showed a significant correlation with pocket depth and jaw bone loss.
Daubert et al. (371)	Patients with dental implants (*n =* 96; 48 males, 48 females; mean age at follow-up = 67.6 ± 10.6 years), of which 5 had DM at implant placement, and 8 had DM at follow-up.	Retrospective cohort study with cross-sectional analysis	*Yes*	Having DM at baseline (placement of dental implants) increased the risk for developing peri-implantitis (RR = 3.0, 95% CI: 1.2–7.7) and for implant loss (RR = 4.8, 95%: 1.8–12.9)
Ferreira et al. (372)	Group 1: Patients with DM (unspecified type, *n =* 29) Group 2: Patients without DM (*n =* 183).	Cross-sectional study	*Yes*	The prevalence of peri-implantitis was significantly increased in patients with DM (24%) compared to the patients without DM (7%).
Gomez-Moreno et al. (373)	Group 1: Patients with HbA_1c_ ≤ 6% (*n =* 21; 9 males, 12 females; mean age = 60 ± 7.2 years). Group 2: HbA_1c_ 6.1–8% (*n =* 24; 11 males, 13 females; mean age = 59 ± 8.1 years). Group 3: HbA_1c_ 8.1–10% (*n =* 11; 6 males, 5 females; mean age = 62 ± 6.8 years). Group 4: HbA_1c_ ≥10.1 (*n =* 11; 7 males, 4 females; mean age = 64 ± 5.6 years).	Cross-sectional study	*Unclear*	Bleeding on probing was significantly different between the groups, with the lowest values in group 1 and the highest values in group 4. Jaw bone loss and pocket depth did not significantly differ between the groups.
Monje et al. (374)	7 epidemiological studies—including 2 prospective cohort studies, 1 retrospective cohort study and 4 cross-sectional studies—were found suitable for meta-analysis (patients with DM: combined *n =* 348, patients without DM: combined *n =* 1,165).	Systematic review and meta-analysis	*Yes*	Patients with hyperglycemia had an almost 50% higher risk for developing peri-implantitis, compared to normoglycemic controls (RR = 1.46, 95% CI: 1.21–1.77).

*T1DM, Diabetes Mellitus Type 1; T2DM, Diabetes Mellitus Type 2; TMJ, Temporomandibular Joint; BMS, Burning Mouth Syndrome; AP, Apical Periodontitis; AGE, Advanced Glycation Endproducts*.

#### Temporomandibular Disorders (TMD)

Temporomandibular disorder (TMD) is an umbrella term that comprises several conditions affecting the temporomandibular region. The principal clinical feature of TMD is pain during mastication, often accompanied by joint sounds and/or limited opening of the jaw. It is estimated that ~10% of the adult population suffers from TMD (375). Earlier in this report, we mentioned that patients with DM are susceptible for joint disorders, primarily through the damaging effects of AGEs on connective tissue. From that perspective, the temporomandibular joint could be affected as well. One study found that the prevalence of temporomandibular joint (TMJ) dysfunction was increased in patients with DM (354). More interestingly, the latter investigators also found that within the diabetic group, peripheral diabetic neuropathy was an independent risk factor for TMJ dysfunction. In a recent animal study, morphologic changes in the TMJ and the capillaries of the retrodiscal tissue were compared between diabetic rats and normal rats. In the diabetic rats, the articular disc of the TMJ was significantly thinner at several sites, compared to the control rats. Also, the capillaries of the retrodiscal tissue were significantly decreased in diameter (355). These findings suggest that TMD might be associated with DM, possibly with involvement of microvascular damage. Research into the pathogenic pathways (hyperglycemia, insulin resistance, dyslipidemia, hypertension, and immune dysfunction) should confirm this hypothesis.

#### Burning Mouth Syndrome

Burning mouth syndrome (BMS) is a chronic pain syndrome. Usually, two types are distinguished: primary (i.e., idiopathic) BMS if there are no underlying diseases that can explain the complaints, and secondary BMS, when there is an oral or systemic disorder that could explain the condition. In the literature, many synonyms for BMS are used (e.g., glossodynia, sore tongue, or stomatodynia), which complicates estimations regarding epidemiology. BMS rarely affects individuals younger than 30, and estimations of the total prevalence range from 0.7 to 4.6% (376). A prospective cohort study from the US found an incidence rate of 11.4 per 100,000 person-years, and showed a strong association with female gender and age (377). Despite the major impact BMS has on quality of life, the pathogenesis is unknown to a large extent. The general complaint of patients with BMS is pain, more specifically a *burning, scalding, tingling*, or *numb* feeling at one or more sites in the mouth, such as the tongue, lip, and hard palate (376). Although pain is the principle complaint, patients with BMS also often experience xerostomia, dysgeusia, and other sensory disorders in the oral region. Also, candidiasis is more prevalent in individuals with BMS, and often causes a burning sensation of the oral mucosa (376). Apparently, there is significant overlap with other oral conditions and complaints that are associated with DM as well. This overlap, together with a lack of a uniform definition of BMS, complicates epidemiologic research into the association with DM. Increased prevalence of BMS in patients with DM compared to healthy subjects has been reported (354, 356), while others did not find any differences in prevalence of BMS (358). In one study, within the people suffering from both DM and BMS, a significant association of BMS with peripheral neuropathy was observed (357). This could indicate that BMS is another symptom of neuropathy in patients with DM, although this theory remains to be confirmed.

#### Pulp Necrosis and Apical Periodontitis

Dental pulp necrosis is the death of cells and tissue inside the root canals and pulp chamber, which can be either symptomatic or asymptomatic. The effect of DM on the dental pulp has mainly been investigated in animal studies. Both acute and chronic effects of hyperglycemia on dental pulp were observed in diabetic rats, characterized by increased inflammation and damage to structural components of the dental pulp (360). This was confirmed in another study, where hyperglycemia in rats resulted in increased inflammation in the dental pulp, which impaired healing after pulp capping (362). Expression of RAGE was also increased in diabetic rats, while treatment of cultured dental pulp cells of diabetic rats with AGEs also resulted in increased inflammatory pathways (365). This generally observed inflammatory response increases the risk for pulp necrosis (378). However, the clinical relevance of these findings are questionable, since well-designed studies with human subjects are lacking.

Closely related to pulp necrosis is periapical or apical periodontitis (AP), a condition in which the periodontal ligament and surrounding alveolar bone around the apex of the tooth is affected. AP is the result of an inflammatory response to an infection of the pulp canals, often caused by caries, trauma, or attrition (379). It is estimated that more than 60% of individuals older than 60 years suffer from at least one AP lesion, but it is often asymptomatic (380). Similar to the marginal form of periodontitis as discussed before (section Periodontal Diseases), the driving force of AP is the inflammatory response to microbial pathogens, which—in the case of AP—find their way beyond the tooth apex. Considering the pathologic similarity between AP and periodontitis, it is highly conceivable that AP is also associated with DM. Several narrative reviews conclude that DM is a risk factor for developing AP, and negatively influences endodontic treatment success (378–382). Multiple cross-sectional studies also show an increased prevalence of AP and an increased number of teeth affected by AP in patients with DM, compared to healthy controls (363, 364, 366–369). Interestingly, two prospective treatment studies showed that treatment of AP is less successful in patients with T2DM, compared to healthy controls (359, 361). Considering the high prevalence of AP, and its similarities to periodontitis, it is rather surprising that so little is known about a possible relationship with DM. Longitudinal studies investigating this relationship should confirm whether DM is a risk factor for the development and severity of AP.

#### Peri-implant Disease

Peri-implant diseases are inflammatory lesions in the tissues around dental implants. As in periodontal diseases, two entities of the condition are recognized: peri-implant mucositis and peri-implantitis (383). Peri-implant mucositis can be compared with gingivitis, affecting the soft tissues surrounding the implant, while peri-implantitis corresponds with periodontitis, also affecting the supporting jaw bone (384). Among individuals with dental implants, the prevalence of peri-implantitis over a 5–15 year period is estimated to range from 15 to 28% (383, 385–388). There is limited epidemiological evidence for an association between DM and peri-implant diseases. Several recent review articles assessed the influence of systemic conditions, among which DM, on the development of peri-implant diseases (389–392). Despite limited evidence of often low quality, it is suggested that poorly controlled DM increases the risk for peri-implantitis, while well-controlled DM is not associated. A systematic review confirmed this, as the meta-analysis of seven studies revealed that patients with hyperglycemia had an almost 50% higher risk for peri-implantitis, compared to normoglycemic subjects (RR = 1.46, 95% CI: 1.21–1.77) (374). On the contrary, one study did not find any differences in jaw bone loss and pocket depth around dental implants between patients with well-controlled and poorly controlled DM. However, it should be noted that the bleeding tendency of the mucosa around implants was associated with higher HbA_1c_ levels (373). A cross-sectional analysis showed a higher prevalence of peri-implantitis in patients with DM, compared to subjects without DM (372). This finding was confirmed in another study, where the researchers found an increased risk for peri-implantitis in patients that suffered from DM at the time of implant placement, compared to patients without DM (RR = 3.0, 95% CI: 1.2–7.7) (371). Interestingly, one study observed a significant correlation between AGEs concentration—measured in peri-implant sulcular fluid and increased in the groups with DM—and pocket depth and jaw bone loss around implants (370).

## Concluding Comments

Prevention and management of well-known complications of DM, such as retinopathy, nephropathy, and neuropathy, are crucial aspects of modern diabetes care. In order to achieve the same for oral complications, this review has provided diabetes care professionals with extensive background knowledge about potential oral complications that can occur in patients with DM. A considerable body of evidence suggests that several oral complications are more prevalent in patients with DM, including periodontitis, dry mouth, dental caries, oral candida infections, oral cancer, and taste disorders. In the case of periodontitis and oral cancer, there are even longitudinal studies that show a temporal association. Some studies suggest that the pathogenic pathways that cause microvascular complications of DM also seem to be involved in oral complications. Even though this is not so evident for all oral complications we discussed, their generally increased prevalence cannot be ignored. Often, there is a lack of decent research that prevents us from establishing or rejecting clear associations between DM and oral diseases and conditions. Therefore, thorough, well-designed research is necessary.

Considering the major impact of oral complications on quality of life, prevention and early management of oral pathologies in diabetes care practice will be crucial. Although this review has provided some insight, recognizing signs and symptoms of oral complications will remain a major challenge for diabetes care professionals. Therefore, as is already the case for the well-known diabetic complications, we strongly encourage an interdisciplinary approach of DM care professionals together with the dental field professionals, to manage potential oral complications.

## Author Contributions

MV, BL, VG, and WT contributed to the conception and drafting of the review. MV and WT reviewed the literature.

### Conflict of Interest Statement

The authors declare that the research was conducted in the absence of any commercial or financial relationships that could be construed as a potential conflict of interest.
